# Reactive power planning based on a proposed voltage stability index in power systems with renewable energy resources

**DOI:** 10.1038/s41598-026-39508-1

**Published:** 2026-04-02

**Authors:** M. Sonbol, Omar H. Abdalla, Abdulla M. Shaheen, Hady H. Fayek

**Affiliations:** 1 Faculty of Engineering, Helwan University, Cairo, Egypt; 2 Faculty of Engineering, Suez University, Suez, Egypt; 3 Faculty of Engineering and Technology, Egyptian Chinese University, Cairo, Egypt

**Keywords:** Index terms—sequential reactive power planning, Identification of the weakest bus, Weakest bus allocation, Voltage stability index and voltage stability improvement, Engineering, Energy grids and networks

## Abstract

*In this paper*, a Reactive Power Planning (RPP) approach based on a proposed voltage stability index is introduced to allocate and size reactive power compensators. The proposed index is employed to identify the weakest bus for compensator placement, while the compensator rating is determined using a sensitivity-based approach to satisfy reactive power requirements under normal operating conditions, (*N* − 1) contingency scenarios, and light-load conditions. Unlike many existing voltage stability indices that rely on solving vector or differential equations and computing Jacobian matrices, the proposed index is formulated as a simple algebraic expression and is efficiently evaluated using a single load-flow solution implemented in tools such as MATLAB script. Based on the proposed index, a sequential RPP framework is developed to systematically allocate and size Static VAr Compensators (SVCs) in order to enhance the system voltage profile and maintain all bus voltages within permissible limits, while achieving the minimum required SVC capacity and the corresponding minimum overall investment cost. The performance of the proposed RPP methodology is benchmarked against metaheuristic optimization techniques, namely Particle Swarm Optimization (PSO) and Grey Wolf Optimization (GWO). A comprehensive performance assessment is conducted using quantitative indices related to voltage profile improvement and economic performance. The proposed voltage stability index is validated using the standard IEEE 9, 14, and 39-bus test systems. The sequential RPP strategy is applied to modified IEEE 14 and 39-bus systems incorporating renewable energy sources such as hydro, wind, and photovoltaic (PV) solar generation. The comparative results, including voltage profile enhancement and cost analysis, demonstrate that the proposed RPP approach effectively compensates reactive power requirements, achieves improved voltage profile and minimum investment costs. All simulation studies are carried out using DIgSILENT PowerFactory and MATLAB.

## Introduction

Reactive Power Planning is one of the main objectives in the modern power system network to allocate the reactive power compensators and determine the compensators rating. In literature, several studies have discussed methods for allocating reactive power sources by detecting weak buses where compensators should be installed^1^. One of this method is the voltage stability margin, which is defined as the distance between the base-load operating point and the voltage collapse point. The weakest bus in the system is the one associated with the minimum voltage stability margin^2^. Various bus voltage stability indices and line stability indices have been proposed to identify candidate buses for compensation^3^. Conventional methods still use the Q–V curve to estimate the reactive power margin, measuring the voltage stability margin^4^ from the base load up to the nose point of the capability curve^5^. The P–V curve can be obtained by gradually increasing the load from the base case until load flow divergence occurs, indicating voltage collapse^6^. The minimum point of the P–V curve corresponds to the weakest bus. Sensitivity-based approaches are also effective for identifying buses most sensitive to reactive power changes^7^. Other techniques use the Jacobian matrix with extensive differential Eq^8^.. Proximity indicators consider both active and reactive power flows to predict the voltage collapse point and assess loading stress on transmission lines. These methods rely on the generalized ABCD parameters of the network, which represent the transmission line accurately^9^. The Continuation Power Flow (CPF) method identifies the voltage collapse point using an augmented Jacobian, thereby avoiding singularity and continuing power flow analysis until the critical point is reached^10^. Modal analysis determines the eigenvalues and eigenvectors of the reduced Jacobian matrix^11^. Artificial intelligence techniques, such as Artificial Neural Networks (ANNs)^12^ and Fuzzy Logic Algorithms (FLA)^13^, have also been applied to determine optimal compensator placement. Moreover, several multi-objective functions^14^, reactive power planning methods^15^, and optimization techniques^16^ have been proposed for optimal reactive power planning. These include analytical, arithmetic programming, and metaheuristic algorithms^17^. Among these, PSO^18^ is one of the most widely used due to its simplicity and efficiency. Other optimization methods include Newton’s method, Successive Quadratic Programming (SQP), Linear Programming (LP)^19^, Nonlinear Programming (NLP)^20^, and Dantzig–Wolfe and Lagrange Relaxation Decomposition Methods (DWDM). Evolutionary Algorithms (EAs)^21^ are also employed for their simplicity, randomness, and solution diversity, although they do not guarantee an optimal solution in finite time and require parameter tuning. Similarly, Genetic Algorithms (GAs)^22^ provide high randomness and diversity, helping to avoid local optima, but they suffer from slow convergence and no guarantee of global optimality.

This research proposes a reactive power planning method using a sequential approach and a proposed voltage stability index to identify the weakest bus for compensator placement. The proposed index is derived from the power capability curve, formulated through a quadratic equation of bus voltage. The method is validated using standard IEEE 9, 14, and 39-bus systems, as well as modified versions incorporating synchronous generators and renewable energy sources. The results are analyzed and compared under both normal and contingency (N–1) operating conditions. The subsequent sections are organized as follows: Sect. “Proposed voltage stability index formulation” presents the formulation of the proposed voltage stability index, while Sect. “Verification of the proposed index” discusses its validation. Section “Test system results and discussion” describes the test systems. Section “Sequential reactive power planning” introduces the proposed sequential method for RPP. Section “SVC model” describes SVC modelling and cost. Section “Data model and simulation results” presents simulation results. Finally, Sect. “Conclusion” summarizes the main conclusions.

## Proposed voltage stability index formulation

**Fig. 1:**
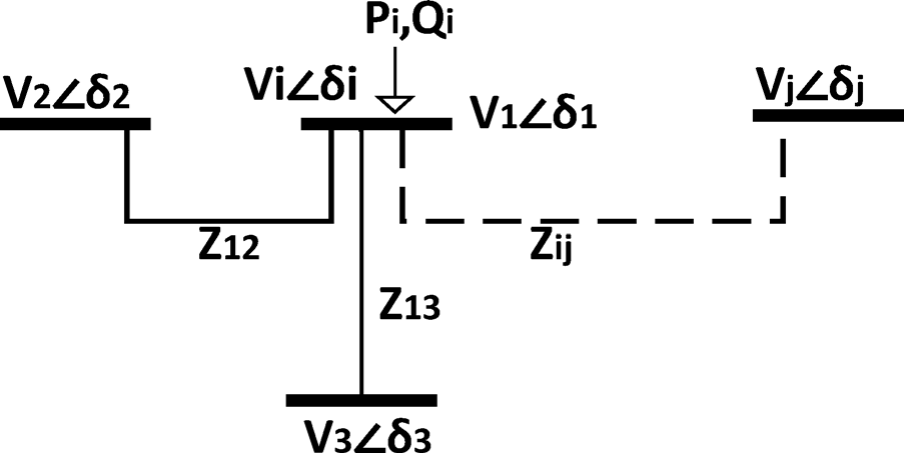
One line diagram of two-bus system.

The proposed index is evaluated for a specific bus *i* (the evaluated bus), which is interconnected with another bus *j* through transmission lines characterized by impedances $\:Zij$. Depending on the network topology, bus *i* may be connected to one or multiple neighboring buses ($\:j=\mathrm{1,2},\:\dots\:\:\mathrm{e}\mathrm{t}\mathrm{c}.$) where $\:i\:\ne\:\:j$ as shown in Fig. 1.

### $\:{V}_{i}$

Voltage at the evaluated bus *i.*

### $\:{V}_{j}$

Voltage at the connected bus *j*, where$\:j=\mathrm{1,2},\:\dots\:.Nc$.

### $\:{N}_{c}$

Number of connected bus to the evaluated bus *i*.

### $\:{\delta\:}_{i}$

Voltage angle at the evaluated bus *i*.

### $\:{\delta\:}_{j}$

Voltage angle at the connected bus *j*.

### $\:{\delta\:}_{ij}$

Angle difference between the voltage angle at the evaluated bus *i* and the voltage angle at the connected bus *j*.

$\:{Z}_{ij}$,$\:{\:Z}_{ik}$: Transmission line impedance between bus *i* and bus *j* or *k*.

$\:{R}_{ij}$,$\:{\:X}_{ij}$: Transmission line resistance and reactance respectively.

### $\:{S}_{i}$

Apparent power injected into the evaluated bus to the transmission line.

$\:{P}_{i}$,$\:{\:Q}_{i}$: Active and reactive power injected into the evaluated bus to the transmission lines respectively.

### $\:{I}_{i}$

Injected current into evaluated bus *i* to the transmission line.

### $\:{I}_{ij}$

Current transfer from bus *i* to the bus *j* through the transmission line *ij.*

LFS: Load flow solution.

1 $$\:{I}_{ij}=\frac{{V}_{i}{\angle\:}\delta\:i-{V}_{j}{\angle\:}\delta\:j}{{R}_{ij}+{JX}_{ij}}$$

Where: $$\:i=1$$ & $$\:i\:\ne\:\:j$$ & $$\:j=\mathrm{2,3},4,\:\dots\:\:{N}_{c}$$

2 $$\:{I}_{i}=\frac{{S}_{i}^{\mathrm{*}}}{{V}_{i}^{\mathrm{*}}}=\frac{{P}_{i}-{JQ}_{i}}{{V}_{i}^{\mathrm{*}}}$$

The injected current $\:{I}_{i}$ at the evaluated bus *i* flowing into the connected transmission line can be calculated from the node bus ($\:i=1$):

3 $$\:{I}_{i}={I}_{12}+{I}_{13}+{I}_{14}+\dots\:.+{I}_{ij\:\:\:\:\:\:\:where\:i{\ddagger}j}$$

Equation (3) can be reformulated by substituting Eqs. (1) and (2) as follows:

$$\:\frac{{S}_{i}^{\mathrm{*}}}{{V}_{i}^{\mathrm{*}}}=\frac{{V}_{1}{\angle\:}\delta\:1-{V}_{2}{\angle\:}\delta\:2}{{Z}_{12}}+\frac{{V}_{1}{\angle\:}\delta\:1-{V}_{3}{\angle\:}\delta\:3}{{Z}_{13}}+\dots\:.+\frac{{V}_{i}{\angle\:}\delta\:i-{V}_{j}{\angle\:}\delta\:j}{{Z}_{ij}}\:where\:i{\ddagger}j$$

Multiply both sides by ($\:{Z}_{12}x{Z}_{13}\dots\:x{Z}_{\mathrm{i}\mathrm{j}})\:$to yields:

$$\:{S}_{i}^{\mathrm{*}}x{Z}_{12}x{Z}_{13}x\dots\:.{Z}_{ij}={V}_{i}^{\mathrm{*}}\{\left({V}_{i}-{V}_{2}\right)x{Z}_{13}x...{Z}_{ik}$$

4 $$\:+{V}_{i}^{\mathrm{*}}\{\left({V}_{i}-{V}_{3}\right)x{Z}_{12}x...{Z}_{ik}+{V}_{i}^{\mathrm{*}}\{\left({V}_{i}-{V}_{j}\right)x{Z}_{12}x{Z}_{13}...{Z}_{ik\:}$$

The left-hand side of Eq. (4) is reformulated as follows:

5 $$\:{S}_{i}^{\mathrm{*}}x{Z}_{12}x{Z}_{13}x\dots\:.{Z}_{ij}={S}_{i}^{\mathrm{*}}x\prod\:_{j=1\&i{\ddagger}j}^{Nc}{Z}_{ij}\:where\:i{\ddagger}j$$

6 $$\:let:\:A+JB=\prod\:_{j=1\&i{\ddagger}j}^{Nc}{Z}_{ij}$$

Equation (5) can be reformulated by substituting Eqs. (2) and (6) as follows:

$$\:{S}_{i}^{\mathrm{*}}x\prod\limits_{j=1\&i{\ddagger}j}^{Nc}{Z}_{ij}=\left({P}_{i}-{JQ}_{i}\right)\left(\:A+JB\right)=$$

7 $$\:\left({P}_{i}-J{Q}_{i}\right)\left(A+JB\right)=\left({P}_{i}xA+{Q}_{i}xB\right)+J\left({P}_{i}xB-{Q}_{i}xA\right)\:$$

The right-hand side of Eq. (4) is reformulatedas:

8 $$\:=\sum\:_{j=1\&i{\ddagger}j}^{Nc}\left[\left({V}_{i}^{\mathrm{*}}x{V}_{i}-{V}_{i}^{\mathrm{*}}x{V}_{j}\right)x\prod\:_{k=1\&i{\ddagger}j{\ddagger}k}^{Nc}{Z}_{ik}\:\right]\:\:\:\:\:\:\:\:\:\:\:\:\:\:\:\:\:\:\:\:\:\:\:\:\:\:\:\:\:\:\:\:\:\:\:\:\:\:\:\:\:\:\:\:\:\:\:\:$$

9 $$\:\:\:\:\:\:\:\:\:\:\:\:\:\:\:\:\:\:\:\:{V}_{i}^{\mathrm{*}}x{V}_{i}=\left|{V}_{i}\right|\:(\mathrm{cos}\delta\:ij+J\mathrm{sin}\delta\:ij\left)\mathrm{x}\left|{V}_{i}\right|\:\right(\mathrm{cos}\delta\:ij-J\mathrm{sin}\delta\:ij)={V}_{i}^{2}\:\:$$

$$\:{V}_{i}^{\mathrm{*}}x{V}_{j}={V}_{i}{\angle\:}\left(-\delta\:i\right)x{V}_{j}{\angle\:}\left(\delta\:j\right)={V}_{i}\:{V}_{j}{\angle\:}\left(\delta\:ji\right)\:,\:\mathrm{W}\mathrm{h}\mathrm{e}\mathrm{r}\mathrm{e}\:\delta\:ji=\delta\:j-\delta\:i$$

10 $$\:{V}_{i}\:{V}_{j}{\angle\:}\left(\delta\:ji\right)={V}_{i}\:{V}_{j}(\mathrm{cos}\delta\:ji+J\mathrm{sin}\delta\:ji)$$

Equation (8) is reformulated by substituting Eqs. (9) and (10) as follows:

11 $$\:\:\:={V}_{i}^{2}\sum\;_{j=1\&i{\ddagger}j}^{Nc}\:\left[\prod\:_{k=1\&i{\ddagger}j{\ddagger}k}^{Nc}{Z}_{ik}\right]-{V}_{i}\sum\:_{j=1\&i{\ddagger}j}^{Nc}\:\left[{V}_{j}(\mathrm{cos}\delta\:ji+J\mathrm{sin}\delta\:ji)\prod\:_{k=1\&i{\ddagger}j{\ddagger}k}^{Nc}{Z}_{ik}\right]$$

Let:

12 $$\:C+JD=\sum\:_{j=1\&i{\ddagger}j}^{Nc}\:\left[\prod\:_{k=1\&i{\ddagger}j{\ddagger}k}^{Nc}{Z}_{ik}\right]\:$$

Let:

13 $$\:E+JF=\sum\:_{j=1\&i{\ddagger}j}^{Nc}\:\left[\left|{V}_{j}\right|(\mathrm{cos}\delta\:ji+J\mathrm{sin}\delta\:ji)\prod\:_{k=1\&i{\ddagger}j{\ddagger}k}^{Nc}{Z}_{ik}\right]$$

By substituting Eqs. (12) and (13) into Eq. (11), Eq. (11) is rewritten as:

$$\:={V}_{i}^{2}\sum\limits_{j=1\&i{\ddagger}j}^{Nc}\:\left[\prod\limits_{k=1\&i{\ddagger}j{\ddagger}k}^{Nc}{Z}_{ik}\right]-{V}_{i}\sum\limits_{j=1\&i{\ddagger}j}^{Nc}\:\left[\left|{V}_{j}\right|(\mathrm{cos}\delta\:ji+J\mathrm{sin}\delta\:ji)\prod\limits_{k=1\&i{\ddagger}j{\ddagger}k}^{Nc}{Z}_{ik}\right]$$

14 $$\:={\left|{V}_{i}\right|}^{2}(C+JD)-\:\left|{V}_{i}\right|\:(E+JF)$$

The left-hand side of Eq. (7) is equal to the right-hand side of Eq. (14); therefore, their imaginary parts are also equal:

15 $$\:{\:\:\:\:\:\:\:\:\:\:\:\:\:\:\:\:\:\:\:\:\:\:\:\:\:\:\:\:\:\:\:\:\:\:\:\:\:\:\:\:(V}_{i}^{2}xD)-\left|{V}_{i}\right|\:x\left(F\right)+\left({{Q}_{i}xA-P}_{i}xB\right)=0$$

16 $$\:{aV}_{i}^{2}+b\left|{V}_{i}\right|+c=0\:$$

Compare the Eqs. (16,15):

$$\:a=D\:\:,\:\:b=-F,\:\:c={{Q}_{i}xA-P}_{i}xB$$

The discriminate of the quadratic equation can be solved as:

$$\:{b}^{2}-4ac>0$$

Substitution from the coefficient of quadric equation:

$$\:\frac{4D\left({{Q}_{i}xA-P}_{i}xB\right)}{{\left[F\right]}^{2}}<1$$

A, B, D, F are functions of the line impedances and voltages ($\:{V}_{j}$) of buses connected to the evaluated bus. The proposed index represents a bus voltage stability index and is written as:

17 $$\:IPVSI=\frac{4D\left({{Q}_{i}xA-P}_{i}xB\right)}{{\left[F\right]}^{2}}<1$$

Where: IPVSI: Injected Power Bus Voltage Stability Index. The weak bus corresponds to a higher IPVSI value, indicating greater vulnerability. The proposed index, with a value close to unity, serves as a reliable indicator of the voltage collapse point. The following paragraph presents the formulation of the proposed index for two case studies.

***Case Study (A)***:

In this case, the evaluated bus ($\:{V}_{1}$) is connected to two buses ($\:{V}_{2}$, $\:{V}_{3}$) through line impedances ($\:{Z}_{12}$, $\:{Z}_{13}$) respectively, the last equations are substituted and rewritten as following:

6 $$\:A+JB=\prod\:_{j=1\&i{\ddagger}j}^{Nc}{Z}_{ij}=\:{Z}_{12}\:x{Z}_{13}$$

$$A=\:{R}_{13}{R}_{12}-{x}_{13}{x}_{12}\:\:\&\:\:B={R}_{13}{x}_{12}+{R}_{12}{x}_{13}$$

12 $$\:C+JD=\sum\:_{j=1\&i{\ddagger}j}^{Nc}\:\left[\prod\:_{k=1\&i{\ddagger}j{\ddagger}k}^{Nc}{Z}_{ik}\right]=\:{Z}_{12}+{Z}_{13}$$

$$\:\mathrm{D}=\:{x}_{12}+{x}_{13}\:\:$$

13 $$\:\:\:\:\:\:\:\:\:\:\:\:\:\:\:\:\:\:\:\:E+JF=\sum\:_{j=1\&i{\ddagger}j}^{Nc}\:\left[\left|{V}_{j}\right|(\mathrm{cos}\delta\:ji+J\mathrm{sin}\delta\:ji)\prod\:_{k=1\&i{\ddagger}j{\ddagger}k}^{Nc}{Z}_{ik}\right]$$

$$\:E+JF={V}_{2}x{Z}_{13}(\mathrm{cos}{\delta\:}_{21}+J\mathrm{sin}{\delta\:}_{21})+{V}_{3}x{Z}_{12}(\mathrm{cos}{\delta\:}_{31}+J\mathrm{sin}{\delta\:}_{31})\:\:\:\:\:\:\:\:\:\:\:\:\:\:\:\:\:\:\:\:\:\:\:\:\:\:\:\:\:\:\:\:\:\:\:\:$$

$$\:E+JF={V}_{2}x{(R}_{13}+\mathrm{J}{x}_{13}\left)\right(\mathrm{cos}{\delta\:}_{21}+J\mathrm{sin}{\delta\:}_{21})+{V}_{3}x{(R}_{12}+\mathrm{J}{x}_{12}\left)\right(\mathrm{cos}{\delta\:}_{31}+J\mathrm{sin}{\delta\:}_{31})\:\:\:$$

$$F=\:{V}_{2}({x}_{13}\mathrm{cos}{\delta\:}_{21}+{R}_{13}\mathrm{sin}{\delta\:}_{21})+{V}_{3}({x}_{12}\mathrm{cos}{\delta\:}_{31}+{R}_{12}\mathrm{sin}{\delta\:}_{31})$$

By substituting the terms, A, B, D and F into Eq. (17), the proposed index is formulated as follows:

$$\:IPVSI=\frac{4D\left({{Q}_{i}xA-P}_{i}xB\right)}{{\left[F\right]}^{2}}$$

17 $$\:IPVSI=\frac{4({x}_{12}+{x}_{13})\left({{Q}_{1}\left({R}_{13}{R}_{12}-{x}_{13}{x}_{12}\right)-P}_{1}({R}_{13}{x}_{12}+{R}_{12}{x}_{13})\right)}{{\left[{V}_{2}({x}_{13}\mathrm{cos}{\delta\:}_{21}+{R}_{13}\mathrm{sin}{\delta\:}_{21})+{V}_{3}({x}_{12}\mathrm{cos}{\delta\:}_{31}+{R}_{12}\mathrm{sin}{\delta\:}_{31})\:\right]}^{2}}$$

Since angle difference $\:\delta\:ji$ is very small value, usually less than 5 deg at worst case under stressed condition, therefore:$\:\mathrm{cos}\delta\:ji\cong1\:\:\:\&\:\mathrm{\:sin}\delta\:ji\cong0$

The approximation affects only the term (F), so the proposed index is written with this approximation as follows:

18 $$\:IPVSI=\frac{4({x}_{12}+{x}_{13})\left({{Q}_{1}\left({R}_{13}{R}_{12}-{x}_{13}{x}_{12}\right)-P}_{1}({R}_{13}{x}_{12}+{R}_{12}{x}_{13})\right)}{{\left[\left({V}_{2}{x}_{13}+{V}_{3}{x}_{12}\right)\right]}^{2}}$$

***Case Study (B)***:

In this case, the evaluated bus (V1) is connected to three buses ($\:{V}_{2}$, $\:{V}_{3},\:{V}_{4}$) through line impedances ($\:{Z}_{12}$, $\:{Z}_{13},\:{Z}_{14}$) respectively, the last equations are substituted as following:

6 $$\:A+JB=\prod\:_{j=1\&i{\ddagger}j}^{Nc}{Z}_{ij}=\:{Z}_{12}\:x{Z}_{13}x{Z}_{14}\:$$

$$\:C+JD=\sum\:_{j=1\&i{\ddagger}j}^{Nc}\:\left[\prod\:_{k=1\&i{\ddagger}j{\ddagger}k}^{Nc}{Z}_{ik}\right]=\:{Z}_{14}{Z}_{12}+{Z}_{13}{Z}_{12}+{Z}_{13}{Z}_{14}$$

12 $$\:\:\:\:\:\:\:\:\:\:\:\:\:\:\:\:\:\:\:\:\:\:\:\:\:\:\:\:\:\:\:\:\:\:\:\:\:\:C+JD={Z}_{14}{Z}_{12}+{Z}_{13}{Z}_{12}+{Z}_{13}{Z}_{14}$$

13 $$\:\:\:\:\:\:\:\:\:\:\:\:\:\:\:\:\:\:\:\:E+JF=\sum\:_{j=1\&i{\ddagger}j}^{Nc}\:\left[\left|{V}_{j}\right|(\mathrm{cos}\delta\:ji+J\mathrm{sin}\delta\:ji)\prod\:_{k=1\&i{\ddagger}j{\ddagger}k}^{Nc}{Z}_{ik}\right]$$

$$\:E+JF=$$

$$\:={V}_{2}x{Z}_{13}{Z}_{14}(\mathrm{cos}{\delta\:}_{21}+J\mathrm{sin}{\delta\:}_{21})+{V}_{3}x{Z}_{12}{Z}_{14}(\mathrm{cos}{\delta\:}_{31}+J\mathrm{sin}{\delta\:}_{31})+{V}_{4}x{Z}_{12}{Z}_{13}(\mathrm{cos}{\delta\:}_{41}+J\mathrm{sin}{\delta\:}_{41})$$

By substituting the terms, A, B, D and F into Eq. (17), the proposed index is formulated as follows:

17 $$\:IPVSI=\frac{4D\left({{Q}_{i}xA-P}_{i}xB\right)}{{\left[F\right]}^{2}}$$

Where:

$$\:A=\left({R}_{13}{R}_{12}{R}_{14}-{{R}_{14}x}_{13}{x}_{12}-{{R}_{12}x}_{13}{x}_{14}-{{R}_{13}x}_{14}{x}_{12}\right)$$

$$\:B=\left({R}_{13}{R}_{12}{x}_{14}-{{x}_{14}x}_{13}{x}_{12}+{{R}_{12}{R}_{14}x}_{13}+{{R}_{13}R}_{14}{x}_{12}\right)$$

$$D=\:\left({R}_{13}{x}_{12}+{R}_{12}{x}_{13}\right)+\left({R}_{13}{x}_{14}+{R}_{14}{x}_{13}\right)+({R}_{14}{x}_{12}+{R}_{12}{x}_{14})$$

$$F=\:{V}_{2}\left[\left({R}_{13}{x}_{14}+{R}_{14}{x}_{13}\right)\mathrm{cos}{\delta\:}_{21}+\left({{R}_{13}R}_{14}-{x}_{13}{x}_{14}\right)\mathrm{sin}{\delta\:}_{21}\right]+$$

$$\:+{V}_{3}[\left({R}_{14}{x}_{12}+{R}_{12}{x}_{14}\right)\mathrm{cos}{\delta\:}_{31}+\left({{R}_{14}R}_{12}-{x}_{14}{x}_{12}\right)\mathrm{sin}{\delta\:}_{31}]+$$

$$\:+{V}_{4}[\left({R}_{13}{x}_{12}+{R}_{12}{x}_{13}\right)\mathrm{cos}{\delta\:}_{41}+\left({{R}_{13}R}_{12}-{x}_{13}{x}_{12}\right)\mathrm{sin}{\delta\:}_{41}]$$

When the last approximation is applied, the new index is formulated as follows:

18 $$\:IPVSI=\frac{4D\left({{Q}_{i}xA-P}_{i}xB\right)}{[{{V}_{2}\left({R}_{13}{x}_{14}+{R}_{14}{x}_{13}\right)+{V}_{3}\left({R}_{14}{x}_{12}+{R}_{12}{x}_{14}\right)+{V}_{4}\left({R}_{13}{x}_{12}+{R}_{12}{x}_{13}\right)]}^{2}}$$

The Injected Power Bus Voltage Stability Index (IPVSI) is a simple algebraic equation that is easily computed using a simple MATLAB script. Furthermore, IPVSI requires only one load-flow solution for evaluation, whereas the indices in the survey may require multiple load-flow solutions under different operating conditions. In this research, IPVSI is employed in the proposed sequential RPP methodology.

## Verification of the proposed index

The validation of the proposed index is performed by comparing it with existing indices reported in the literature. The following paragraph presents a review of selected indices for this comparison.

### V/V0 bus voltage stability index

The weakest bus has minimum ratio of $\:\mathrm{V}/{\mathrm{V}}_{\mathrm{o}}$ index^1^. The voltage $\:{V}_{\mathrm{o}}$ can be evaluated at each bus at base case. While the voltage (V) is evaluated at different loading factor (λ) higher than the base load for example (λ = 125%, 150%. etc.)

### Q-V curve

The reactive power margin serves as a measure of the voltage stability at a given bus. It is defined as the range of reactive power from the base load up to the voltage stability limit. At each bus, reactive power is gradually increased until load flow divergence occurs, which indicates the voltage collapse point. This procedure is repeated for all buses, and the maximum reactive power that causes voltage collapse is recorded for each bus. The weakest bus is identified as the bus with the minimum recorded reactive power margin^2^.

### P-V curve

The voltage stability margin is evaluated using the P–V curve. The procedure involves gradually increasing the total network load at a constant power factor until the voltage collapse point is reached. The bus voltages are recorded at each loading condition to identify the weak bus, which corresponds to the minimum point on the P–V curve just before the occurrence of voltage collapse^6^.

## Test system results and discussion

**Fig. 2:**
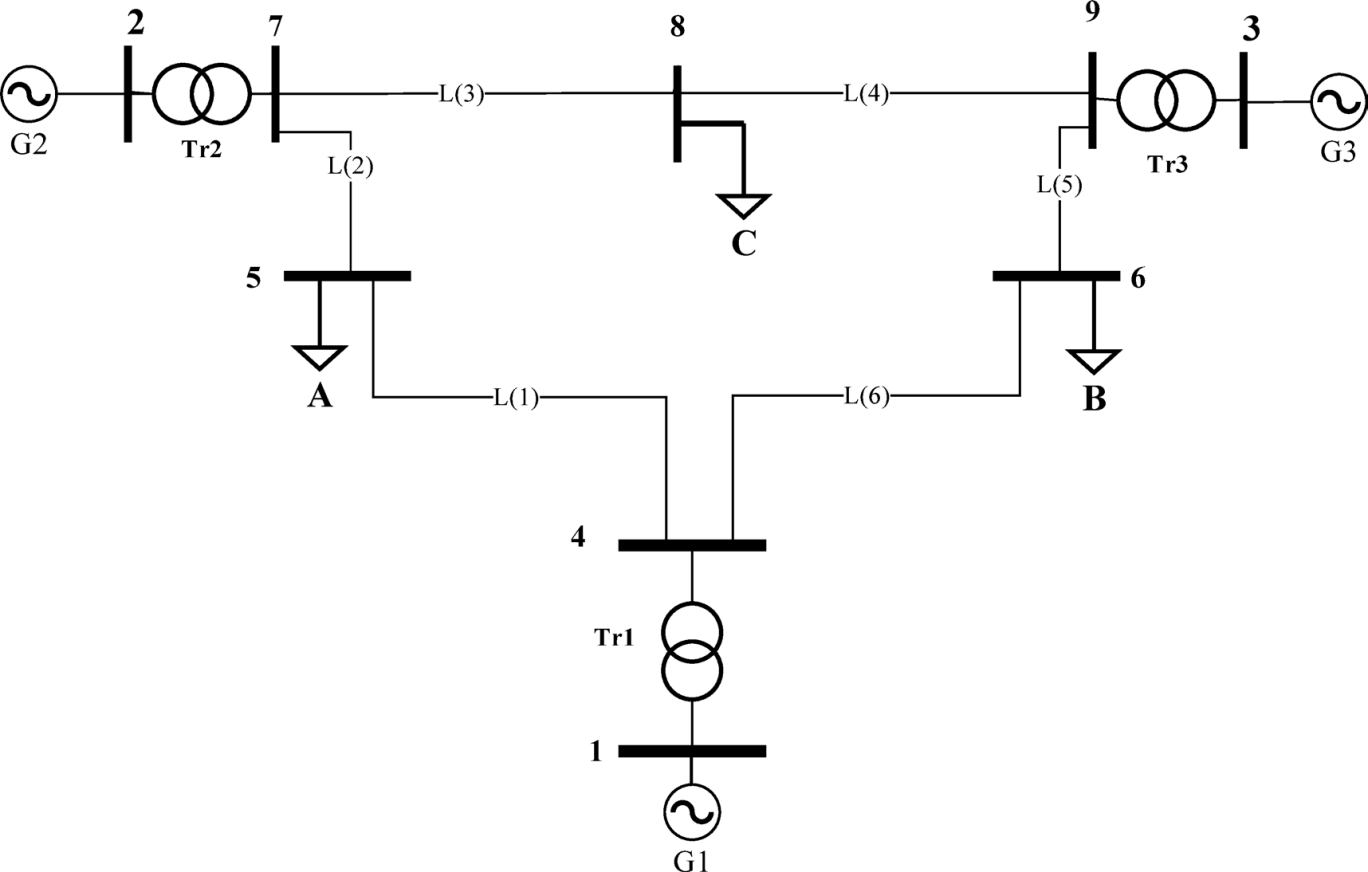
IEEE 9-bus system.

**Fig. 3:**
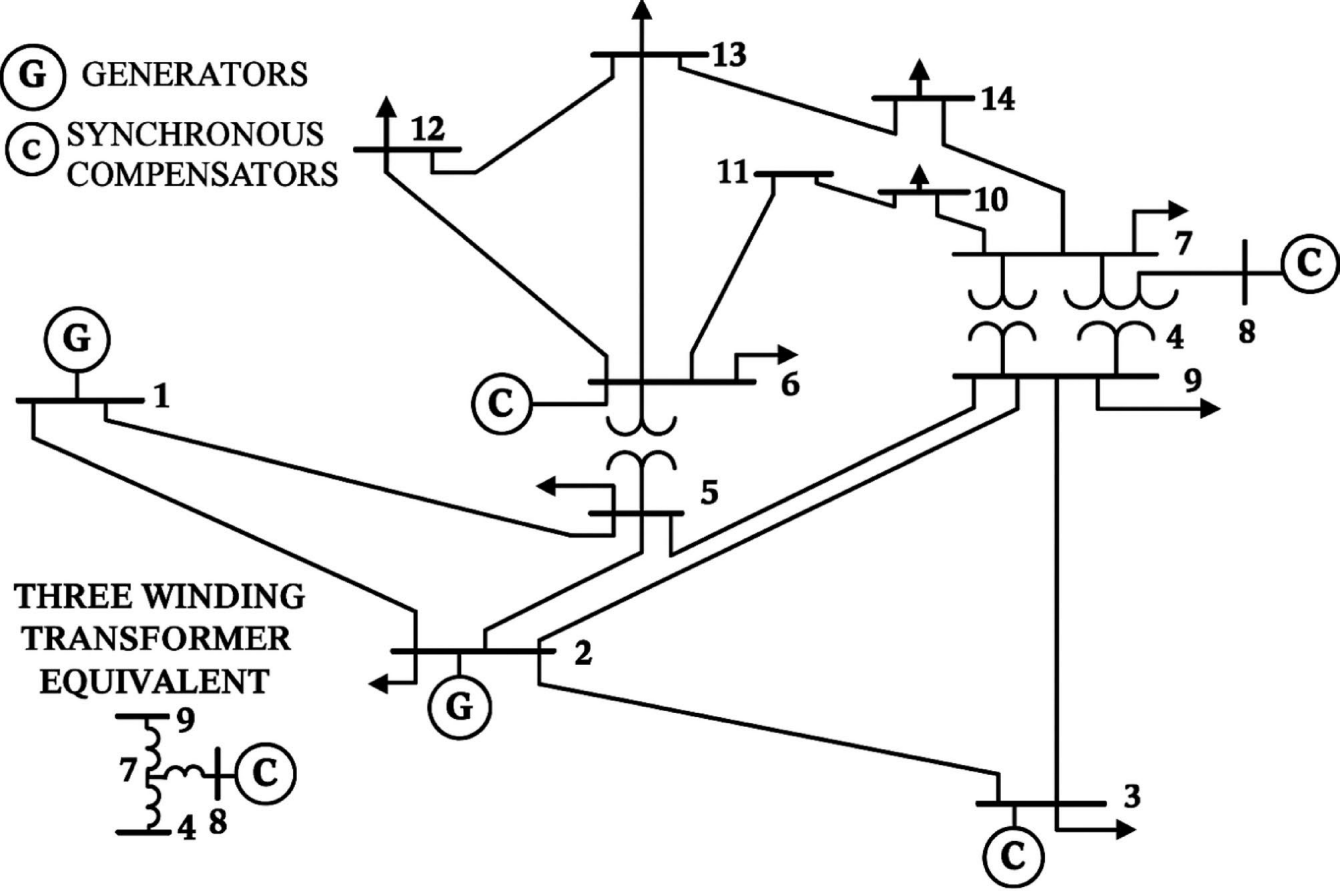
IEEE 14-bus system.

**Fig. 4:**
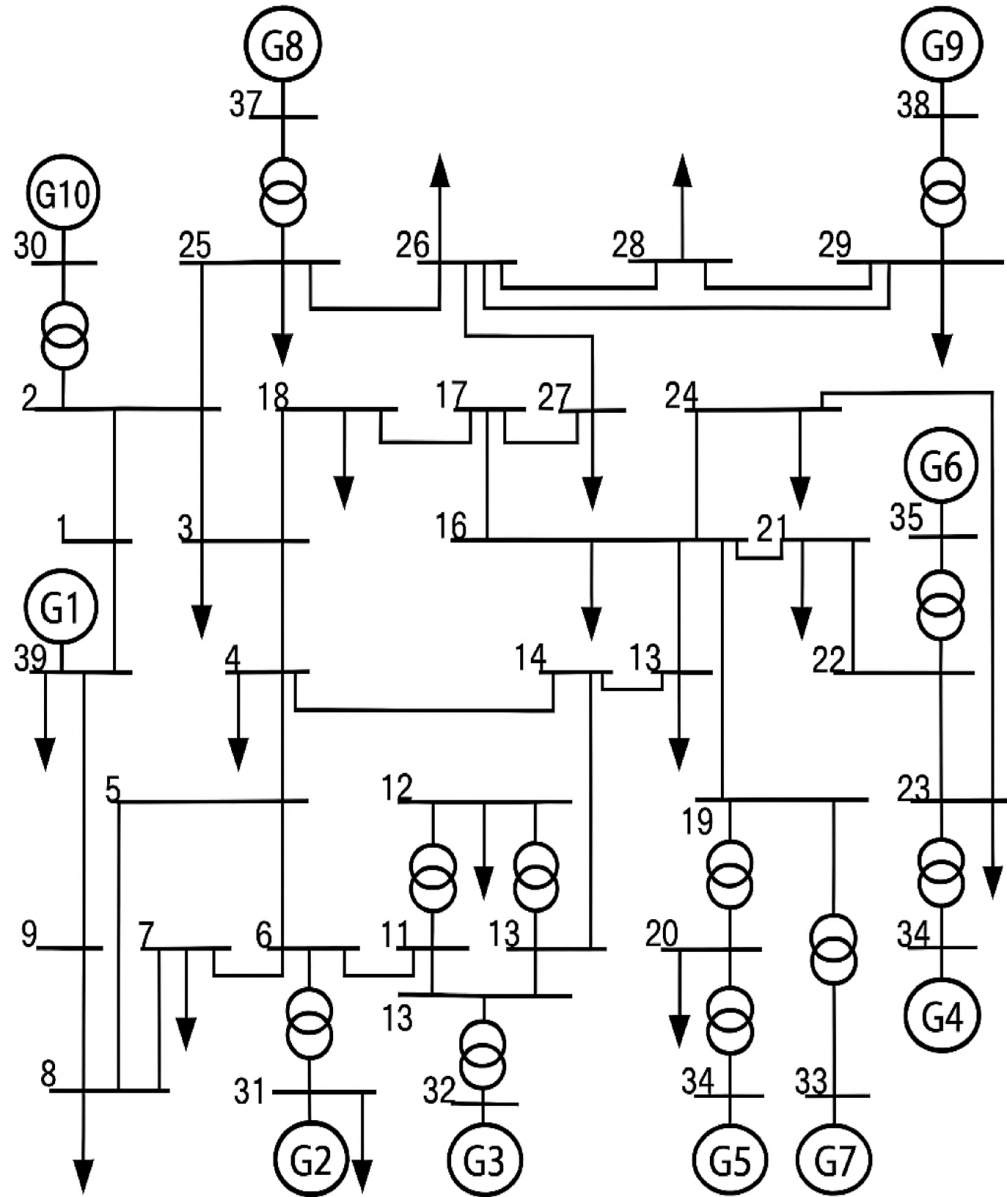
IEEE 39-bus system.

Figures 2, 3 and 4 show the standard IEEE 9-bus^23^, 14-bus^24^ and 39-bus^25^ test systems, respectively, employed to validate the performance of the proposed index in comparison with the existing indices in the literature.

### Results and analysis of V/Vo ratio index

**Fig. 5:**
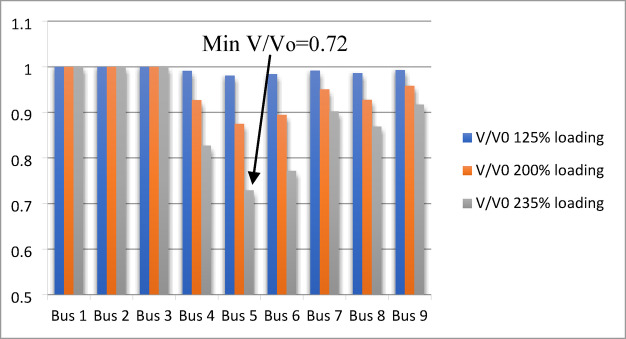
$\:\mathrm{V}/{\mathrm{V}}_{\mathrm{o}}$ index applied for IEEE 9-bus system.

The $\:\mathrm{V}/{\mathrm{V}}_{\mathrm{o}}$ ​index is evaluated at each bus under different loading conditions to identify the weakest bus, which corresponds to the minimum ratio. The IEEE 9-bus system is analyzed at various loading levels (125%, 200%, and 235% of the base load). The $\:\mathrm{V}/{\mathrm{V}}_{\mathrm{o}}$​ index for each bus under these loading conditions is shown in Fig. 5.

**Fig. 6:**
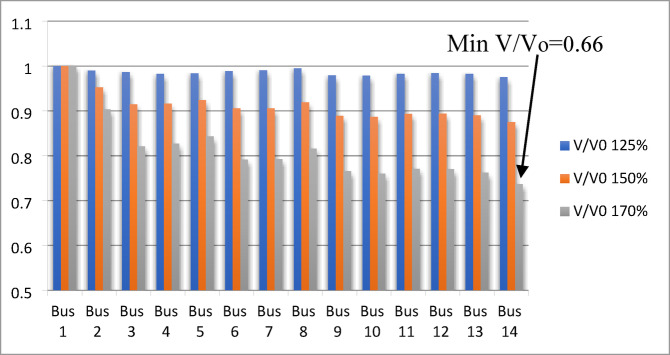
$\:\mathrm{V}/{\mathrm{V}}_{\mathrm{o}}\:$index applied for IEEE 14-bus system.

Bus (5) is identified as the weakest bus with a minimum index value ($\:\mathrm{V}/{\mathrm{V}}_{\mathrm{o}}$=0.72) at 235% loading. Bus 6 follows with a ratio of $\:\mathrm{V}/{\mathrm{V}}_{\mathrm{o}}$= 0.77, and bus 4 with $\:\mathrm{V}/{\mathrm{V}}_{\mathrm{o}}$= 0.82. Thus, the ranking of the weak buses are 5, 6, and 4 respectively. For the IEEE 14-bus system, calculations are performed at loading levels of 125%, 150%, and 170%, as shown in Fig. 6. Minimum index value ($\:\mathrm{V}/{\mathrm{V}}_{\mathrm{o}}$=0.66) at 170% loading for bus 14 as the weakest bus; then, bus 10 with ratio ($\:\mathrm{V}/{\mathrm{V}}_{\mathrm{o}}$=0.6971) and bus 13 with index value ($\:\mathrm{V}/{\mathrm{V}}_{\mathrm{o}}$=0.6972). Thus, the ranking of the weak buses becomes (14,10,13,9,12) respectively.

**Fig. 7:**
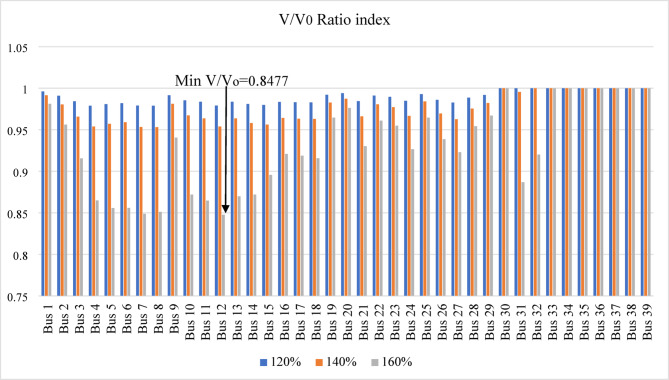
$\:\mathrm{V}/{\mathrm{V}}_{\mathrm{o}}$ index applied for IEEE 39-bus system.

Figure 7 shows the $\:\mathrm{V}/{\mathrm{V}}_{\mathrm{o}}$ ratio of the IEEE 39-bus system, where bus 12 is identified as the weakest bus with a minimum value of 0.8477 at 160% loading. The voltage remains constant under different loading conditions for the generating buses (30–39) at 120% and 140% loading, while the voltages at buses 31 and 32 drop at 160% loading due to exceeding the reactive power limits of generators $\:{G}_{2}$ and $\:{G}_{3}$, respectively.

### Q-V curve

**Fig. 8:**
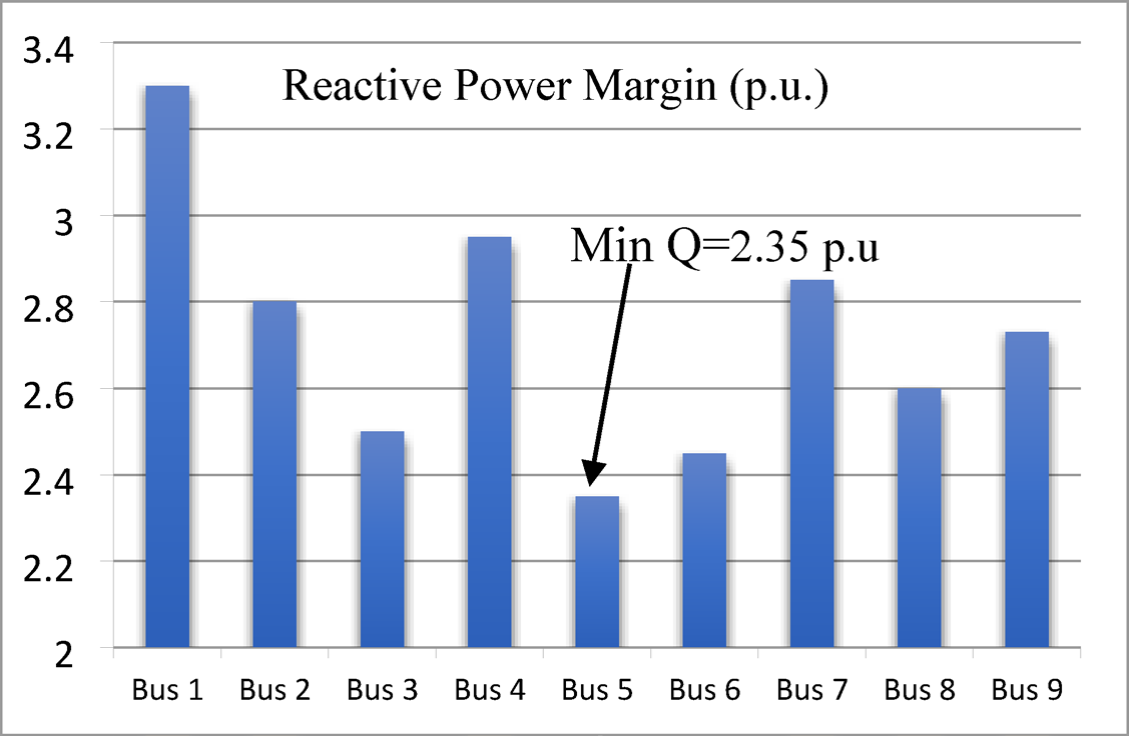
Reactive Power Margin (p.u.) for IEEE 9-bus system.

The reactive power at each bus is gradually increased until divergence is reached, and the maximum reactive power is recorded as the voltage stability margin. This margin is used to identify the weakest bus, which has the minimum reactive power margin. These steps are applied to the standard IEEE 9, 14 and 39-bus systems. The voltage stability margin for the IEEE 9-bus system is shown in Fig. 8. Bus 5 is identified as the weakest bus with a minimum reactive power margin of 2.35 p.u., followed by bus 6 with 2.45 p.u. and bus 3 with 2.50 p.u. Thus, the ranking of the weak buses is 5, 6, 3, and 8, respectively.

**Fig. 9:**
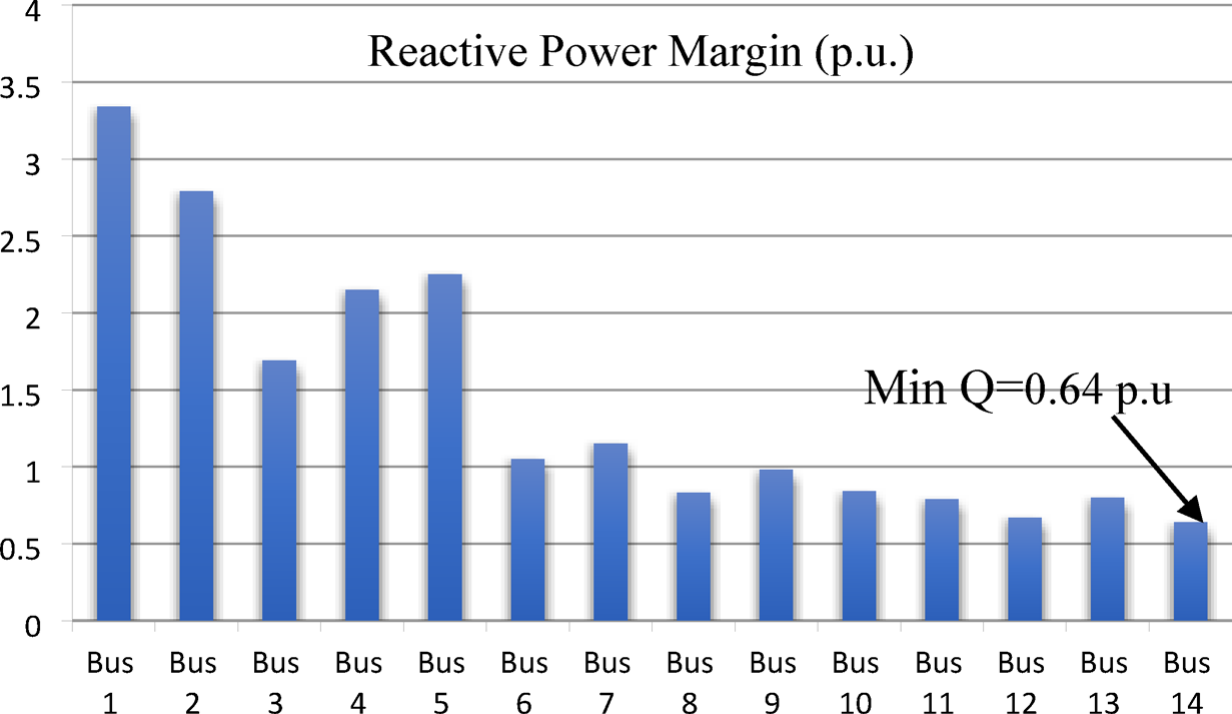
Reactive Power Margin (p.u) for IEEE 14-bus system.

The voltage stability margin for the IEEE 14-bus system identifies bus 14 as the weakest bus with a minimum reactive power margin of 0.64 p.u. as shown in Fig. 9. Bus 12 follows with a margin of 0.67 p.u., while buses 11, 13, and 10 have margins of 0.79, 0.80, and 0.84 p.u., respectively. Thus, the ranking of the weak buses is 14, 12, 13, 11, 8, 10 and 9 respectively.

**Fig. 10:**
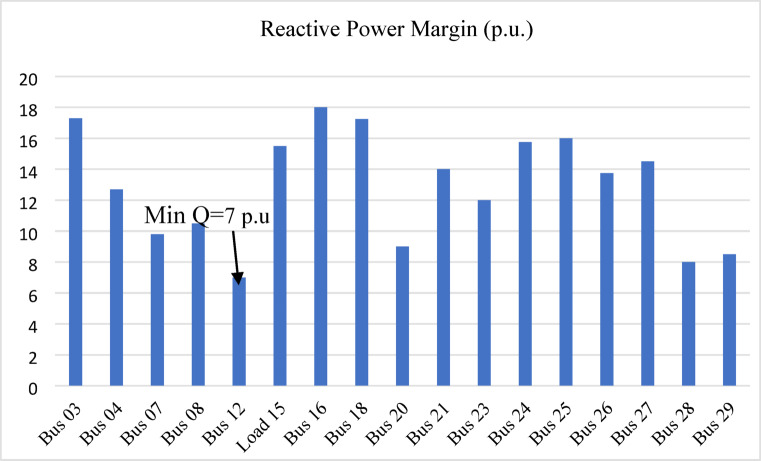
Reactive Power Margin (p.u) for IEEE 39-bus system.

In the IEEE 39-bus system, bus 12 is identified as the weakest bus with a minimum reactive power margin of 7 p.u. as shown in Fig. 10. The generating buses are excluded from the graph because they already supply reactive power and have higher reactive power margins than the load buses.

### P-V curve

**Fig. 11:**
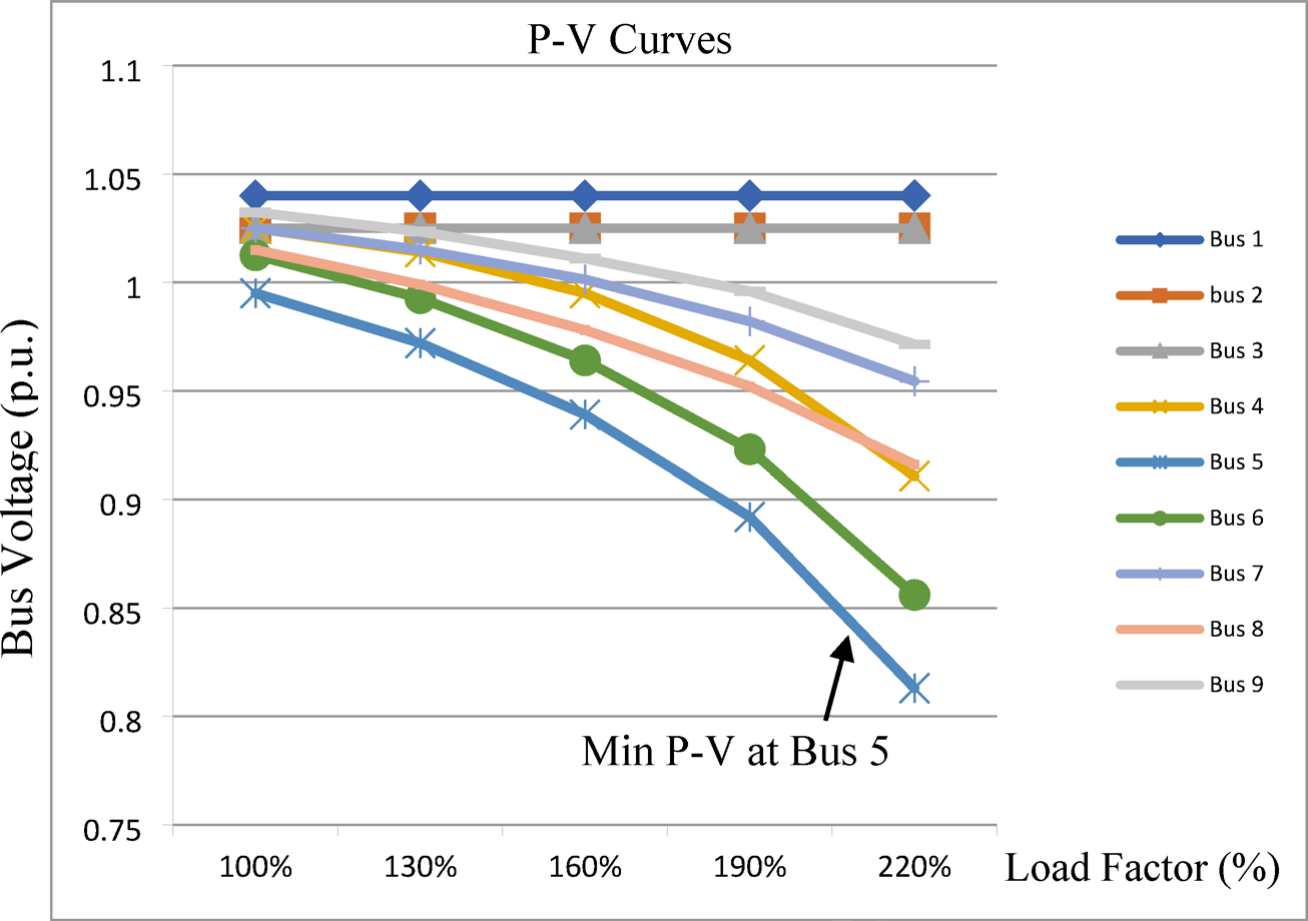
P-V Curves for IEEE 9-bus system.

The P–V curves of the IEEE 9-bus system are obtained by gradually increasing the load factor from 100% to 220% of the base load at a constant power factor, with increments of 30% per step, and recording the bus voltages (p.u.) at each stage as shown in Fig. 11. Bus 5 exhibits the lowest P–V curve and is therefore identified as the weakest bus, followed by buses 6, 8, and 4 respectively.

**Fig. 12:**
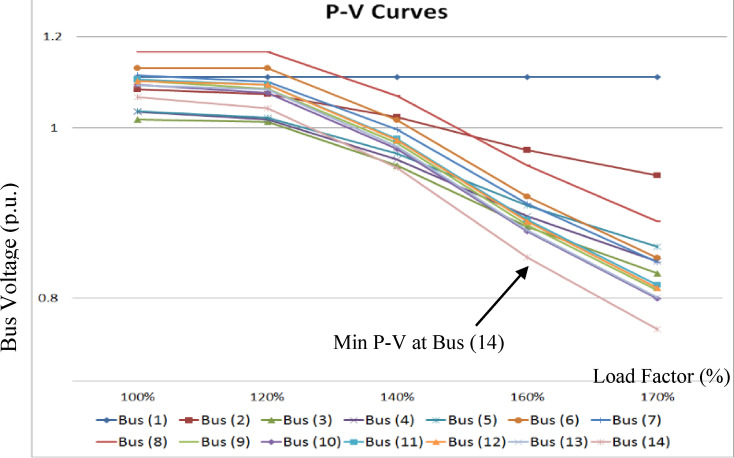
P-V Curves for IEEE 14-bus system with different loading.

**Fig. 13:**
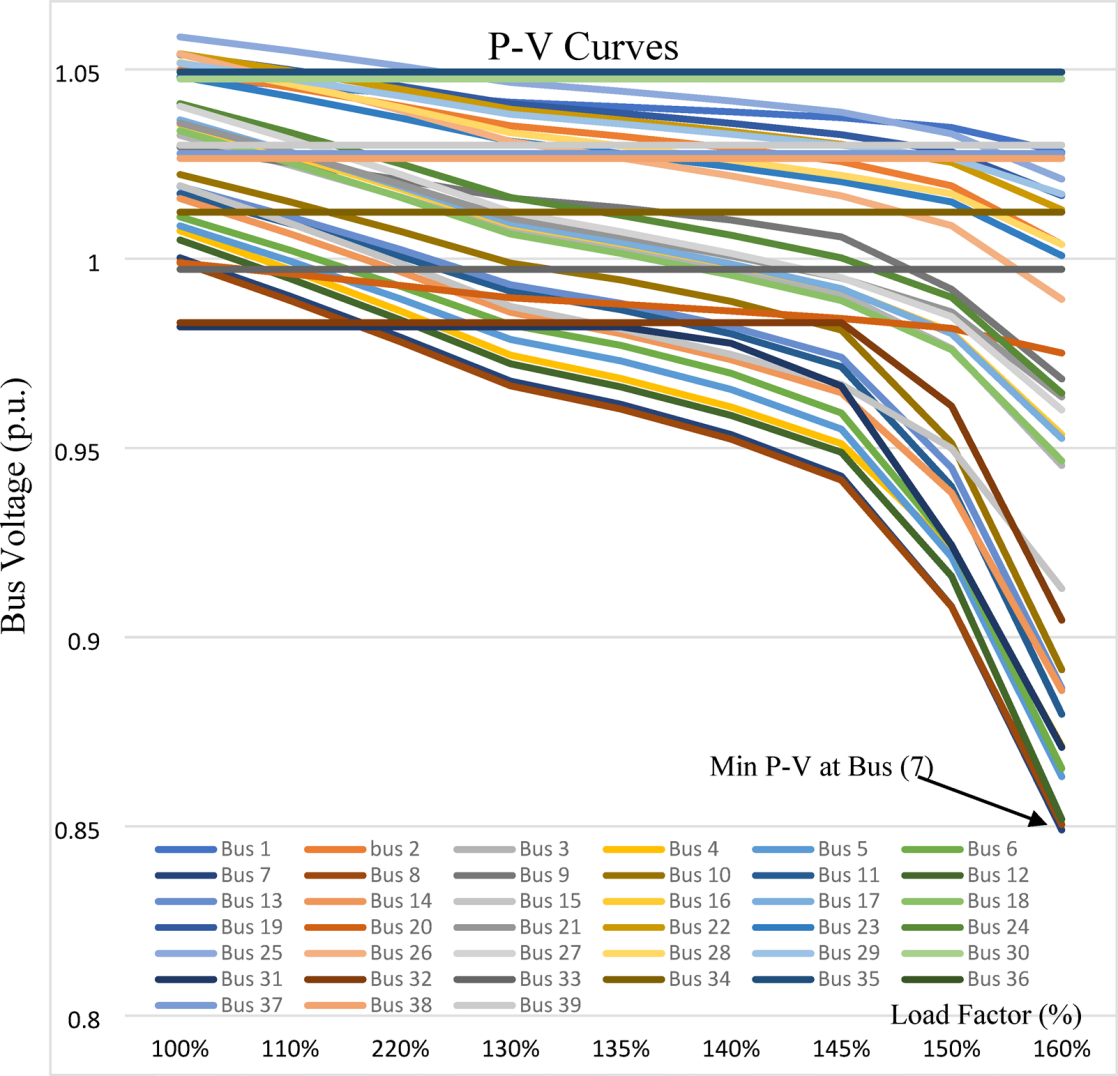
P-V Curves for IEEE 39-bus system with different loading.

For the IEEE 14-bus system, the loads are gradually increased from 100% to 170% of the base load, in increments of 10% per step, and the bus voltages (p.u.) are recorded at each stage that is shown in Fig. 12. Bus 14 exhibits the lowest P–V curve and is therefore identified as the weakest bus, followed by buses 10 and 13, respectively. In the IEEE 39-bus system, bus 7 exhibits the lowest P–V curve and is therefore considered the weakest bus, as shown in Fig. 13.

### Proposed index (IPVSI)

**Fig. 14:**
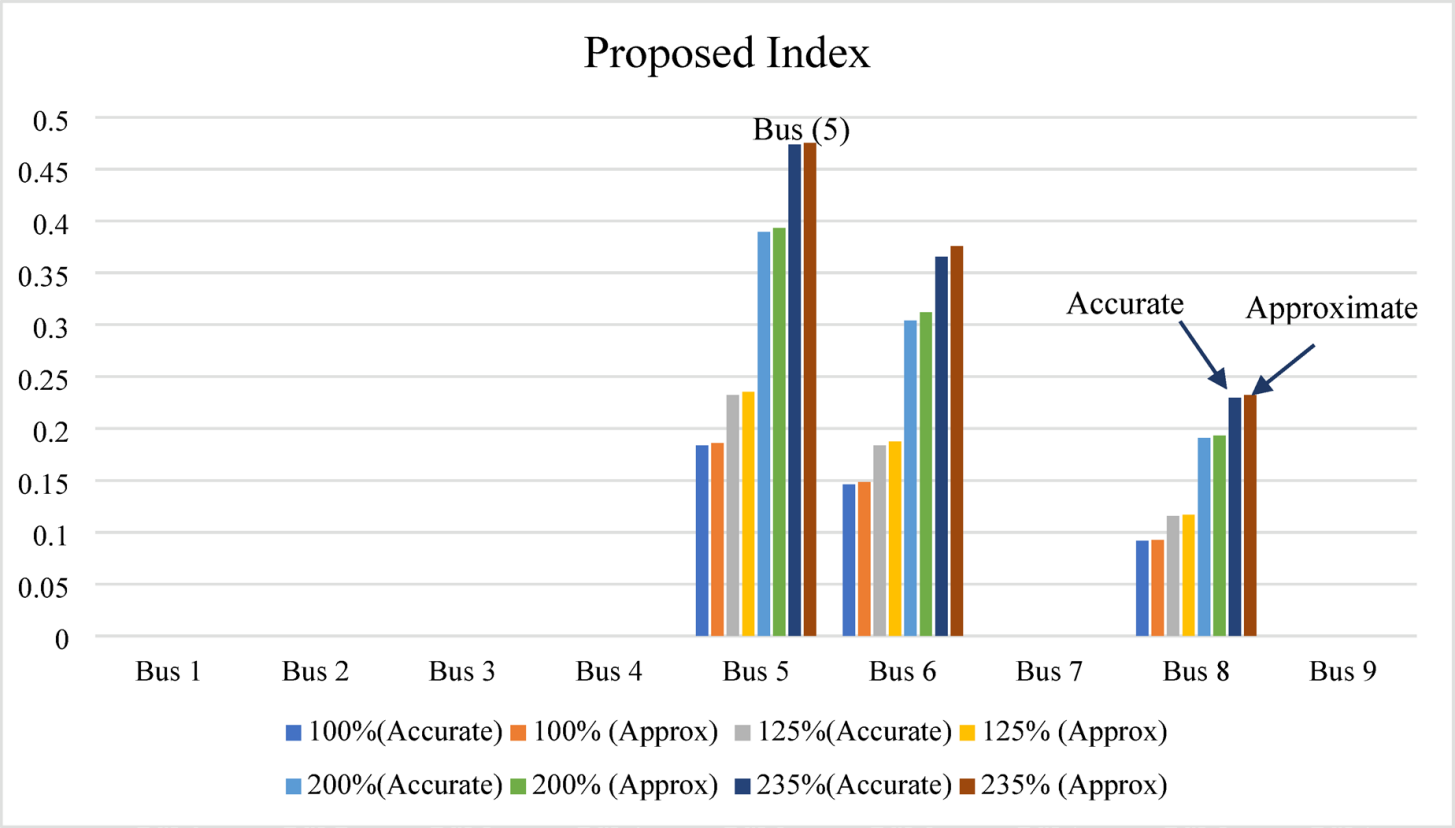
Proposed index result for IEEE 9-bus system.

To evaluate the performance of the proposed index, it is calculated at each bus under different loading conditions, requiring only a single load flow solution for each loading level. The index is applied to the IEEE 9-bus test system at loading levels of 100%, 125%, 200%, and 235%. In all cases, bus 5 is identified as the weakest bus, exhibiting the highest IPVSI value under both base-case and stressed operating conditions. Buses 6 and 8 are identified as the next weakest buses, respectively, as illustrated in Fig. 14. Both the accurate and approximate values decide that bus 5 is the weakest bus at over all scenarios loading conditions. Furthermore, the results at the 235% loading level (stressed condition) demonstrate that the error with value 0.286% between the accurate and approximate IPVSI values (0.47379491 and 0.475154011 respectively) at bus 5 is negligible. Buses equipped with reactive power control exhibit negative IPVSI values due to the injection of capacitive reactive power at the evaluated bus; therefore, these controlled buses are excluded from consideration as weak buses.

**Fig. 15:**
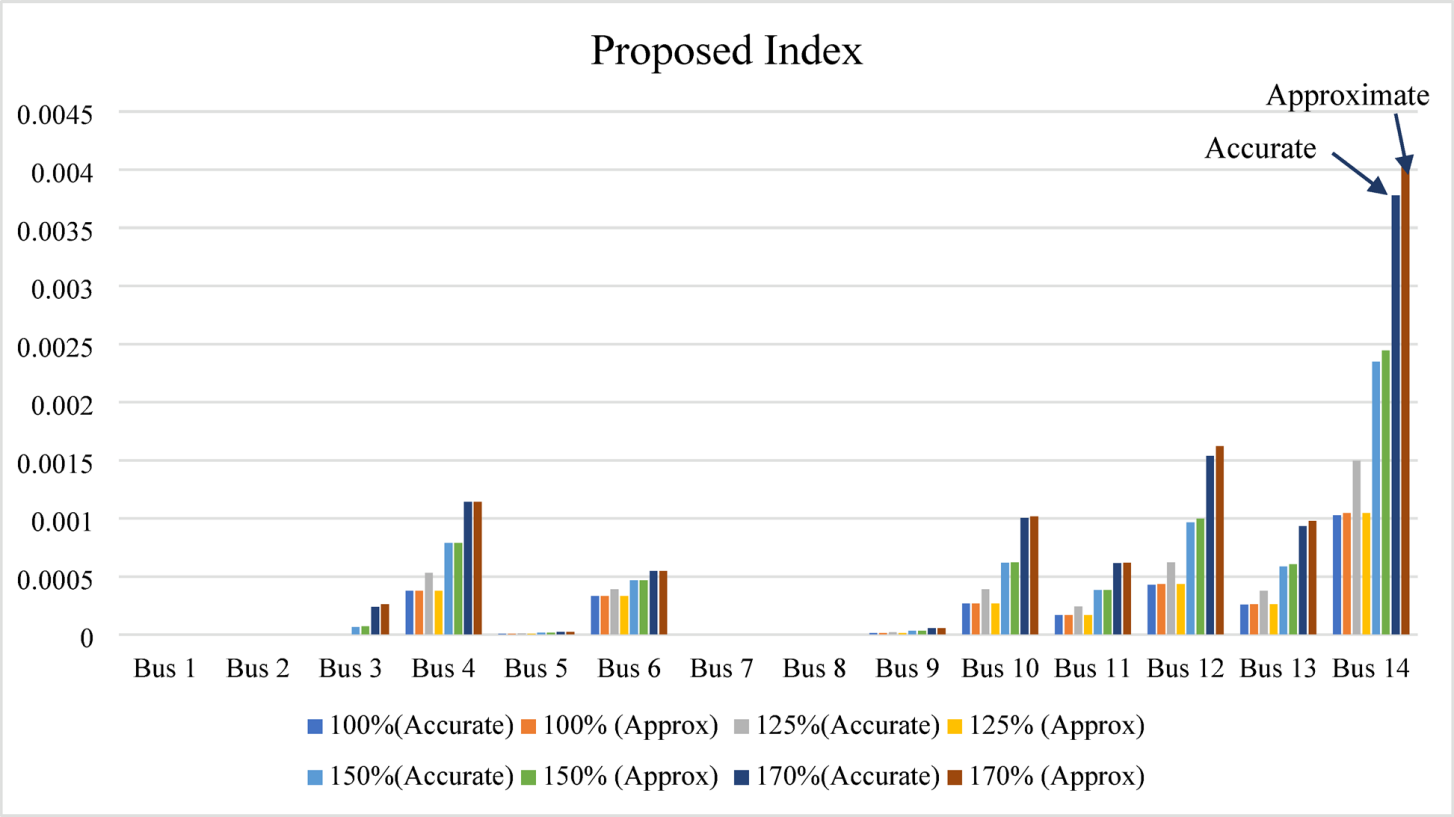
Proposed index result for IEEE 14-bus system.

In the IEEE 14-bus system, bus 14 is identified as the weakest bus, exhibiting a higher IPVSI value than the other buses under all loading conditions (100%, 125%, 150%, and 170%), as shown in Fig. 15. At the 170% loading level, the accurate and approximate IPVSI values at bus 14 are nearly identical (0.003779213 and 0.00403646, respectively), confirming that bus 14 is the weakest bus. This close agreement demonstrates that the IPVSI remains reliable when computed using either the accurate or the approximate formulation.

**Fig. 16:**
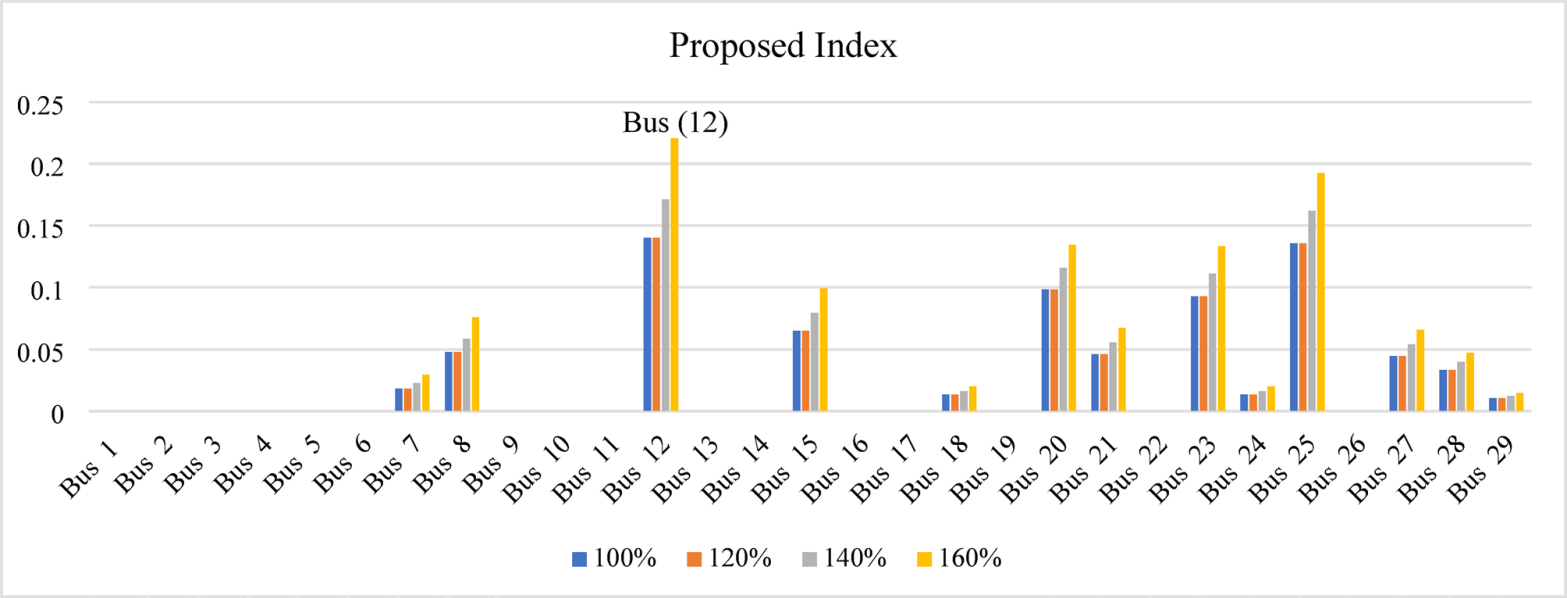
Proposed index result for IEEE 39-bus system.

In the IEEE 39-bus system, the proposed index reaches its maximum value of 0.111 at bus 12 under the base-case condition and increases to 0.220646337 at the 160% loading level. Accordingly, bus 12 is identified as the weakest bus, as shown in Fig. 16.

Tables 1, 2 and 3 summarize the bus voltage stability indices from the literature and their corresponding rankings, compared with the proposed index for the IEEE 9, 14, and 39-bus systems. Table 1 identifies bus 5 as the weakest bus in the IEEE 9-bus system, while Table 2 shows bus 14 as the weakest bus in the IEEE 14-bus system.

In Table 3, the proposed index and two other indices from the literature survey, namely the Q–V and $\:\mathrm{V}/{\mathrm{V}}_{\mathrm{o}}$ ratio indices, identify bus 12 as the weakest bus. It is noted that the P–V curve identifies bus 7 as the weakest bus, which differs from the results obtained using the proposed index and other methods. This discrepancy arises because P–V curves describe the variation of bus voltage with active power, reflecting the system’s ability to transfer real power. In contrast, Q–V curves represent the relationship between voltage and reactive power, directly indicating reactive power reserve and voltage stability limits. Although the “nose point” of both curves signifies voltage collapse, the Q–V curve more explicitly captures reactive power deficiencies, which are critical for voltage support and stability assessment. Accordingly, the other methods identify bus 12 as the weakest bus, as their stability analysis is based on variations in reactive power demand.

The results summarized in Tables 1, 2 and 3 confirm that the proposed index provides a correct ranking of weak buses, consistent with several indices reported in the literature. The proposed index requires only a single load flow solution under normal operating conditions. In contrast, many existing indices require multiple load flow solutions at different loading levels. Moreover, the proposed index is computed using a simple algebraic equation, whereas other indices may rely on differential equations or the Jacobian matrix.

A comprehensive comparison between the proposed index and other indices reported in the literature is presented in Table 4.

**Table 1:** Comparison of bus voltage indices for IEEE 9-bus system.

$\:\mathrm{V}/{\mathrm{V}}_{\mathrm{o}}$ Ratio Index at 200% Loading		Voltage Stability Margin		P-V Curve at 200% Loading		Proposed Index	
Index Value	Weakest Bus No.	$\:{\mathrm{Q}}_{\mathrm{m}\mathrm{a}\mathrm{x}}$ (p.u.)	Weakest Bus No.	Voltage (p.u.)	Weakest Bus No.	Index Value	Weakest Bus No.
0.874	5	2.35	5	0.871	5	0.186904	5

**Table 2:** Comparison of bus voltage indices for IEEE 14-bus system.

$\:\mathrm{V}/{\mathrm{V}}_{\mathrm{o}}$ Ratio Index at 170% Loading		Voltage Stability Margin		P-V Curve at 170% Loading		Proposed Index	
Index Value	Weakest Bus No.	$\:{\mathrm{Q}}_{\mathrm{m}\mathrm{a}\mathrm{x}}$ (p.u.)	Weakest Bus No.	Voltage (p.u.)	Weakest Bus No.	Index Value	Weakest Bus No.
0.736	14	0.64	14	0.682	14	0.000996	14

**Table 3:** Comparison of bus voltage indices for IEEE 39-bus system.

$\:\mathrm{V}/{\mathrm{V}}_{\mathrm{o}}$ Ratio Index at 170% Loading		Voltage Stability Margin		P-V Curve at 160% Loading		Proposed Index	
Index Value	Weakest Bus No.	$\:{\mathrm{Q}}_{\mathrm{m}\mathrm{a}\mathrm{x}}$ (p.u.)	Weakest Bus No.	Voltage (p.u.)	Weakest Bus No.	Index Value	Weakest Bus No.
0.847	12	7.00	12	0.849	7	0.111300	12

**Table 4:** Comprehensive comparison for proposed index vs. conventional methods.

Method	Face Comparison
Proposed Index	¬ The proposed IPVSI is derived directly from steady-state nodal voltage–power equations and does not rely on the Jacobian matrix, sensitivity analysis, or differential equations commonly used in continuation power flow or modal analysis techniques. Furthermore, the formulation avoids vector- and matrix-based computations, resulting in a purely scalar algebraic expression evaluated at each bus. This structure allows the index to be easily computed using basic tools such as simple MATLAB scripts, enhancing its practical applicability for real-time monitoring and planning studies. ¬ The IPVSI formulation does not rely on simplifying assumptions or reduced-order equivalents. In contrast, several existing indices are derived using approximated models, such as two-bus system equivalents, and often assume constant power factor loads or neglect line resistance, shunt elements, or admittance effects. Such approximations may introduce inaccuracies when applied to large-scale, meshed, and renewable-rich power systems. The proposed index fully incorporates actual network parameters and nodal interactions, thereby improving accuracy and robustness ¬ While IPVSI shares a steady-state analytical foundation with other single-load-flow indices, its nodal-based derivation, scalar formulation, and non-reliance on approximations provide a clear theoretical distinction. This distinction is further supported by its consistent performance under both normal and contingency (*N* − 1) operating conditions, as demonstrated in the revised simulation results.
Q-V Curve	¬ Load Flow Solution (LFS): Requires many load flow solutions at different loading conditions. ¬ Calculation Requirements: Computationally intensive calculations. ¬ Computation Speed: Time-consuming, due to the large number of load flow solutions and repeated calculations for all buses
P-V Curve	¬ LFS: Requires many load flow solutions under different loading conditions. ¬ Calculation Requirements: computationally intensive calculations. ¬ Computation Speed: Time-consuming, due to the large number of load flow solutions required.
Line Stability Indices	¬ LFS: Most line stability indices require multiple load flow solutions under varying loading scenarios and extensive comparative analysis to identify weak buses. In contrast, the proposed index requires only a single load flow solution. The resulting index values can be directly interpreted through a simple column chart-based procedure to identify the weakest buses based on their higher index values, significantly reducing computational effort. ¬ Both the L-index and the Fast Voltage Stability Index (FVSI) are line-based stability indices that assume unidirectional power flow from the sending end to the receiving end. This assumption may not hold in modern power systems with distributed generation, bidirectional power flows, and meshed network structures. In contrast, the proposed index is a bus voltage stability index derived directly from nodal voltage equations, enabling it to accurately represent interconnected power networks without relying on directional power flow assumptions. ¬ Conventional line stability indices primarily provide qualitative information by classifying system components as “weak” or “strong,” without explicitly quantifying the Voltage Stability Margin (VSM). Although they can identify critical lines, their capability for precise margin estimation is limited, particularly under multiple contingencies. Conversely, the proposed index quantitatively evaluates the voltage stability margin for each bus under both normal and contingency (*N* − 1) operating conditions. ¬ The performance of line-based indices is highly dependent on the system topology and operating point; an index that performs well under certain conditions may yield misleading results in other scenarios. The proposed index mitigates this limitation by relying on nodal interactions rather than individual line characteristics, thereby maintaining consistent performance across different network configurations and loading conditions.
Other Voltage Stability Indices	¬ Most other voltage stability indices (VSIs) are derived using simplified system representations that assume linear behavior and neglect important components such as line shunt admittances and detailed load characteristics, which can significantly reduce their accuracy when applied to complex, real-world power systems. While, no neglecting for any parameters or assumptions for the proposed index. ¬ Some voltage stability indices neglect critical factors such as reactive power dynamics, generator reactive power limits, and the influence of control devices, all of which are essential for accurately assessing overall voltage stability. ¬ Many voltage stability indices, particularly those based on Jacobian matrices, impose a high computational burden, which limits their suitability for fast or real-time online voltage stability monitoring. ¬ Different voltage stability indices are derived based on diverse mathematical assumptions—such as neglecting certain power flow components or emphasizing specific voltage equations that can lead to inconsistent results and varying performance across different operating scenarios.

## Sequential reactive power planning

**Fig. 17:**
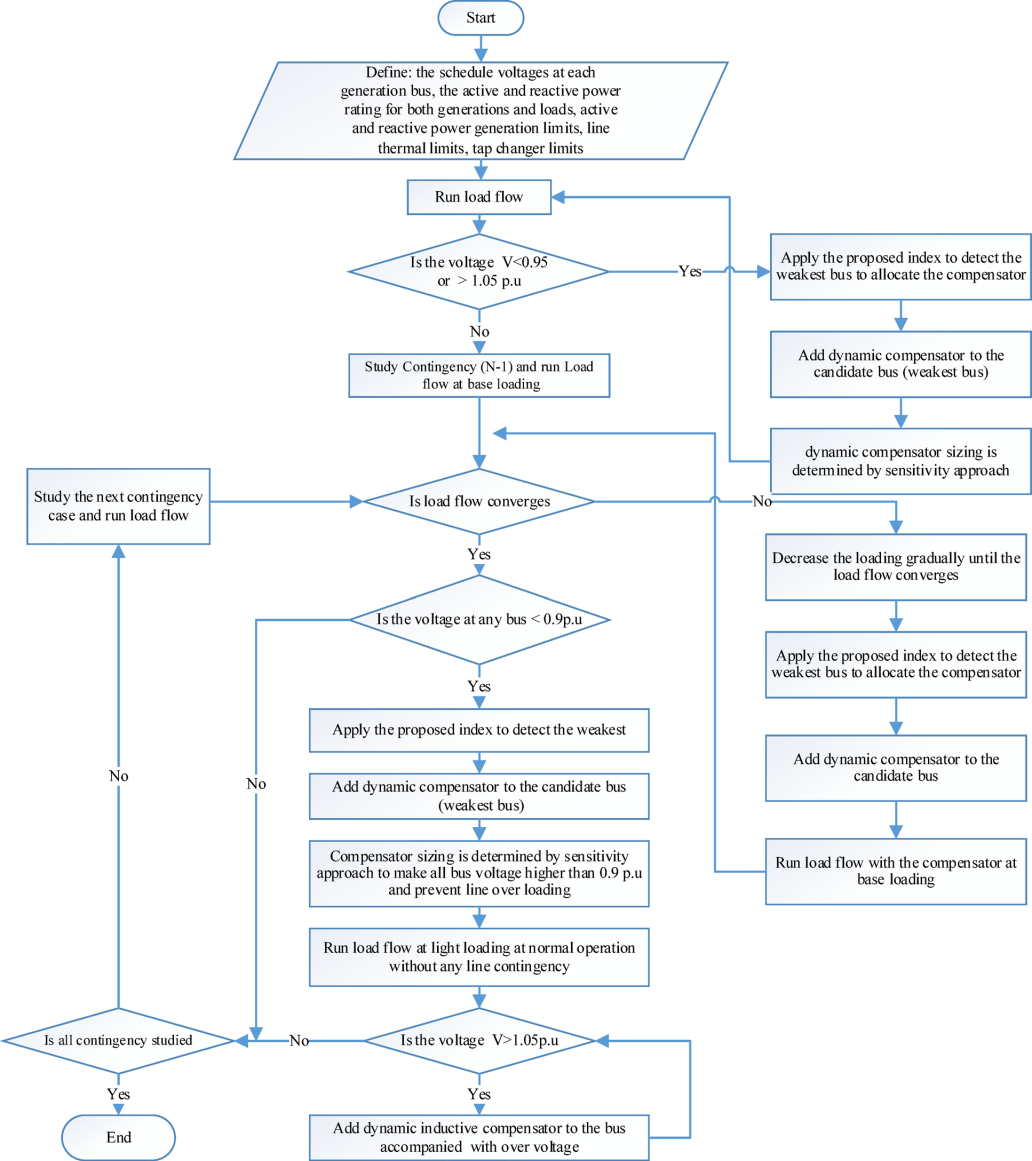
Sequential reactive power planning flow chart.

Figure 17 shows the flowchart of the proposed sequential RPP procedure. The main objective of sequential RPP is the allocation and sizing of reactive power compensators based on the proposed index. The following paragraphs describe the procedure for performing sequential RPP on any power system network.

 1 st step: Define the scheduled voltages at each generator bus, the line impedances, and the active and reactive power at both generation and load buses. Then, perform a load flow analysis under normal operating conditions at the base loading. The following considerations should be carefully observed during RPP:

1. Active power generation limits

$$\:{P}_{g,i}^{\mathrm{m}\mathrm{i}\mathrm{n}}\le\:{P}_{g,i}\le\:{P}_{g,i}^{\mathrm{m}\mathrm{a}\mathrm{x}}\:\:$$

2. Reactive power generation limits are specified according to the generation types and their capability curves

$$\:{Q}_{g,i}^{\mathrm{m}\mathrm{i}\mathrm{n}}\le\:{Q}_{g,i}\le\:{Q}_{g,i}^{\mathrm{m}\mathrm{a}\mathrm{x}}$$

- The reactive power limits of synchronous generator related to the capability curve^26^. The upper reactive power limit $\:{Q}_{g,i}^{\mathrm{m}\mathrm{a}\mathrm{x}}$ is calculated at 0.85 lag power factor at rated active power, while the lower reactive power limit $\:{Q}_{g,i}^{\mathrm{m}\mathrm{i}\mathrm{n}}$ is calculated at 0.95 lead power factor at rated active power.
- The reactive power limits of wind generation related to its capability chart^27^, the power factor of wind farms shall operate from 0.95 lag to 0.95 lead at rated active power. Accordingly, the upper reactive power limit $\:{Q}_{g,i}^{\mathrm{m}\mathrm{a}\mathrm{x}}$ is calculated at 0.95 lag power factor, while the lower reactive power limit $\:{Q}_{g,i}^{\mathrm{m}\mathrm{i}\mathrm{n}}$ is calculated at 0.95 lead power factor.
- The reactive power limits of PV generation depend on the plant scale and the corresponding capability curves. Medium-scale solar plants operate within a power factor range of 0.95 lagging to 0.95 leading at rated active power. Large-scale solar plants can supply reactive power during both daylight and night time hours, operating across all possible points in the P–Q diagram^28^, also within the 0.95 lagging to 0.95 leading power factor range at rated active power.

3. Bus voltage margin

$$\:{V}_{i}^{\mathrm{m}\mathrm{i}\mathrm{n}}\le\:{V}_{i}\le\:{V}_{i}^{\mathrm{m}\mathrm{a}\mathrm{x}}\:$$

4. Line thermal limit

$$\:\left|{S}_{ij}\right|<{S}_{ij}^{\mathrm{r}\mathrm{a}\mathrm{t}\mathrm{i}\mathrm{n}\mathrm{g}}\:\:$$

2nd step: If the bus voltages fall below the permissible limits, the proposed index is used to identify the weakest bus as the candidate location for reactive power compensator allocation. Initially, the compensator is considered as a dynamic device capable of satisfying all contingency cases. The sensitivity approach is then applied to determine the appropriate compensator rating. These steps are repeated iteratively until all bus voltages are maintained within permissible limits under normal operating conditions (0.95p.u. ≤ V ≤ 1.05p.u.)^26^.

3rd step: Next, the contingency scenario (N-1) is considered, and a load flow analysis is performed. The load flow may either converge or diverge. When the load flow converges, the bus voltages should remain within the permissible limits of ± 10%^26^. If the voltage at any bus falls below 0.9 p.u. during contingency, the proposed index is used to identify the candidate bus for compensator allocation. The compensator rating is then determined using the sensitivity approach to ensure that all bus voltages exceed 0.9 p.u. under contingency conditions.

4th step: If the load flow diverges at the base loading during any contingency, the load should be reduced until the load flow converges. The proposed index can then be applied to identify the candidate bus for compensator allocation. Next, the load flow is performed again under the same contingency case with the new compensation at base loading. These steps are repeated iteratively until the load flow converges at base loading under the contingency scenario.

5th step: After implementing the new compensation, the load flow is checked under normal operating conditions (without any contingency) to ensure that all bus voltages remain within permissible limits. These steps are repeated iteratively until all contingency cases, including both line and generator contingencies, have successfully evaluated and addressed.

6th step: Next, the light load case is studied at 40% of the base loading^29^ to evaluate potential overvoltage phenomena at any bus caused by excessive capacitive reactive power compensation. If overvoltage occurs, a dynamic inductive compensator should be installed at the affected bus. Finally, all bus voltages are ensured to be within permissible limits under normal operation, contingency scenarios, and light load conditions. The following paragraphs present the simulation results for the modified IEEE 9, 14, and 39-bus systems.

## SVC model

**Fig. 18:**
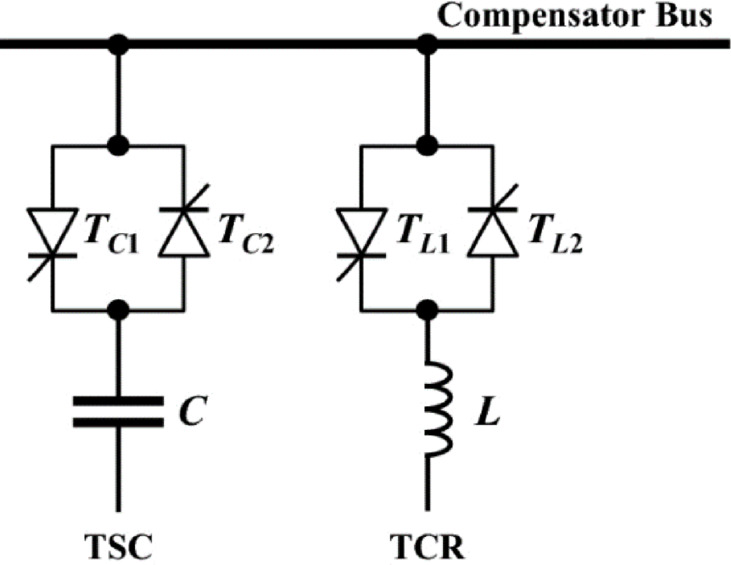
SVC schematic diagram.

The physical feasibility of static VAr compensation is primarily determined by factors such as substation layout availability, system voltage level, short-circuit capacity, and environmental constraints. SVC installations require sufficient space to accommodate thyristor-controlled reactors, thyristor-switched capacitor banks, harmonic filters, cooling systems, and associated control equipment, as shown in Fig. 18. According to IEEE grid code requirements and relevant standards, including IEEE Std 421, IEEE Std 519, and IEEE Std 1547^30^ (where applicable), SVCs are required to provide continuous and dynamic reactive power control within their rated limits. This ensures that bus voltages are maintained within permissible ranges, typically ± 5% of the nominal voltage during normal operation and up to ± 10% during contingency events or voltage disturbances.

### SVC limits

The dynamic limits of the reactive power contribution of the SVC are modeled as:

When SVC operates in inductive mode:

19 $$\:{Q}_{L\:max=}{\left({U}_{rated}\right)}^{2}\times\:{B}_{Lrated}\:$$

When SVC operates in capacitive mode:

20 $$\:{Q}_{C\:max=}{-\left({U}_{rated}\right)}^{2}\times\:{B}_{Crated}\:\:\:\:\:\:\:\:\:\:\:\:\:\:\:\:\:\:\:\:\:\:\:\:\:\:\:\:\:\:\:\:\:\:\:\:\:\:$$

Where, $\:{Q}_{L\:rated}$ and $\:{Q}_{C\:rated}\:$are the rated inductive and capacitive reactive power of SVC (p.u), respectively; $\:{U}_{rated}$ is the rated voltage (p.u); $\:{B}_{Lrated}$ and $\:{B}_{Crated}$ are the susceptance for the inductor and capacitor (p.u), respectively.

### SVC cost model

The investment cost of installing SVC in the system is modeled as follows^20^:

21 $$\:{SVC\_Cost}_{i}={\sum\:}_{i=1}^{{N}_{SVC}}\left({K}_{i}+{C}_{i}\times\:\left|{QSVC}_{i}\right|\right)$$

Where, $\:{K}_{i}$ is the fixed installation cost of SVC at bus i, $\:{N}_{SVC}$ is the number of SVC.

$$\:{C}_{i}$$ is the cost coefficient ($/MVAr).

$\:{QSVC}_{i}$ is the reactive power capacity of the SVC at bus *i* (MVAr).

Recent market summaries and technical studies indicate that the typical installed cost of SVCs ranges from approximately $50 to $150 per kVAr (≈ $50k–$150k per 1 MVAR in 2025), with a commonly cited midpoint of $100/kVAr (~$100k/MVAR). The final installed cost depends on factors such as the system voltage level, project scope, civil works, and control and communication requirements^31^. The constant K is assumed $50k as fixed installation cost for each SVC.

## Data model and simulation results

The sequential RPP approach is applied to the IEEE 14-bus and IEEE 39-bus systems, with specific modifications introduced to the standard test networks to incorporate renewable energy sources. Wind and solar generation units are integrated into the systems, typically exhibiting lower reactive power capability limits than conventional synchronous generators. In addition, selected network configuration adjustments are implemented to prevent load flow divergence under contingency (N–1) conditions. SVCs are installed at the identified weak buses, and their ratings are determined using sensitivity analysis. The following paragraphs describe, in detail, the systematic procedure for the allocation and sizing of reactive power compensators to ensure that all bus voltage magnitudes remain within permissible limits under normal operation, contingency (N–1) scenarios, and light-load conditions.

### Modified IEEE fourteen bus test system

The modified IEEE 14-bus test system retains the standard line and transformer parameters of the original network, as shown in Fig. 3. To simulate a more stressed operating condition, the load levels are increased to 200%. The generation data, incorporating renewable energy sources and relevant assumptions, are summarized in Table 5. In this configuration, three synchronous condensers at buses 3, 6, and 8 are disconnected, and the shunt capacitor at bus 9 is deactivated.

**Table 5:** Generation data of modified IEEE 14-bus system.

Gen Type	Busbar	Bus T.	Voltage Mag (*p*.u.)	Voltage Angle (deg)	Rated Power (MVA)	Lower Limit of Reactive Power (MVAr)	Upper Limit of Reactive Power (MVAr)	Active Power (MW)
Wind Farm (1)	Bus 1	PV	1	---	160	−48	48	150
Photovoltaic (1)	Bus 2	PV	1	---	80	−24	24	75
Wind Farm (2)	Bus 3	PV	1	---	125	−38	38	120
Photovoltaic (3)	Bus 6	PV	1	---	35	−11	11	32
Hydro	Bus 8	SL	1	0	155	−47	80	---

**Fig. 19:**
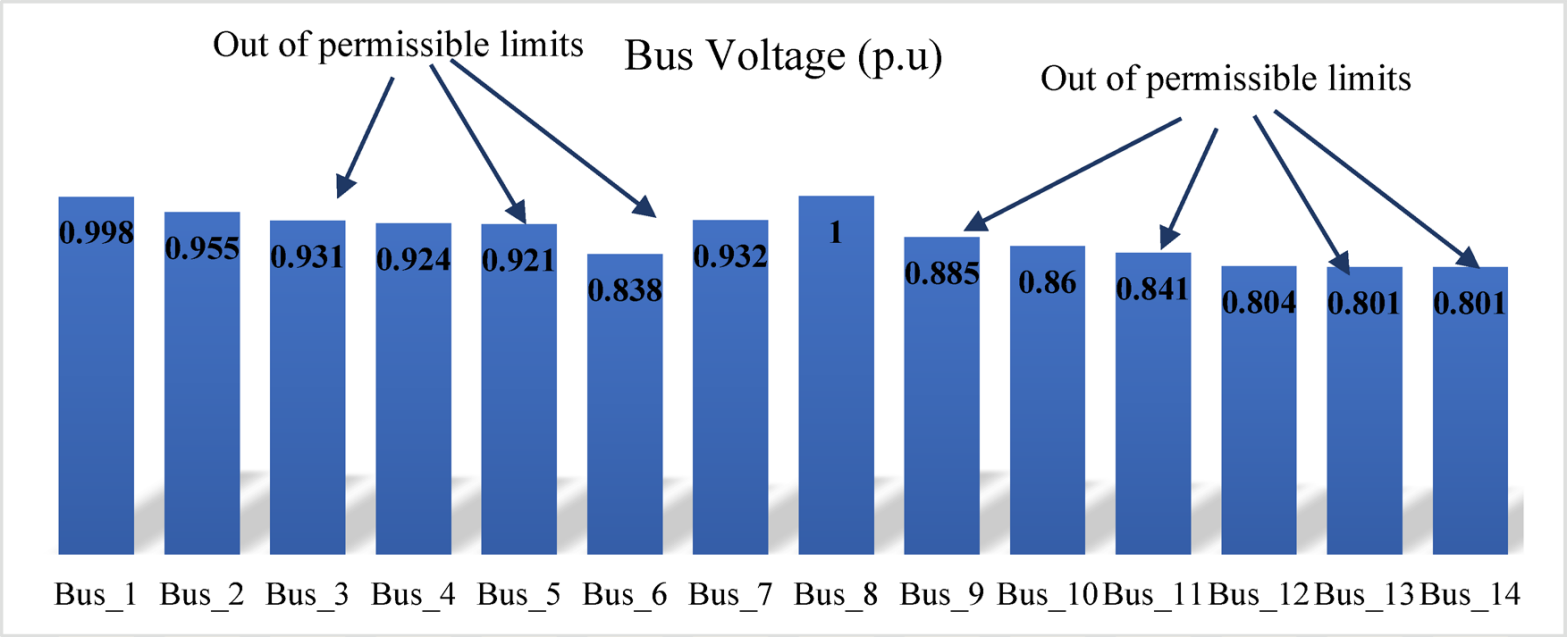
Bus voltages for modified IEEE 14-bus before compensation.

**Fig. 20:**
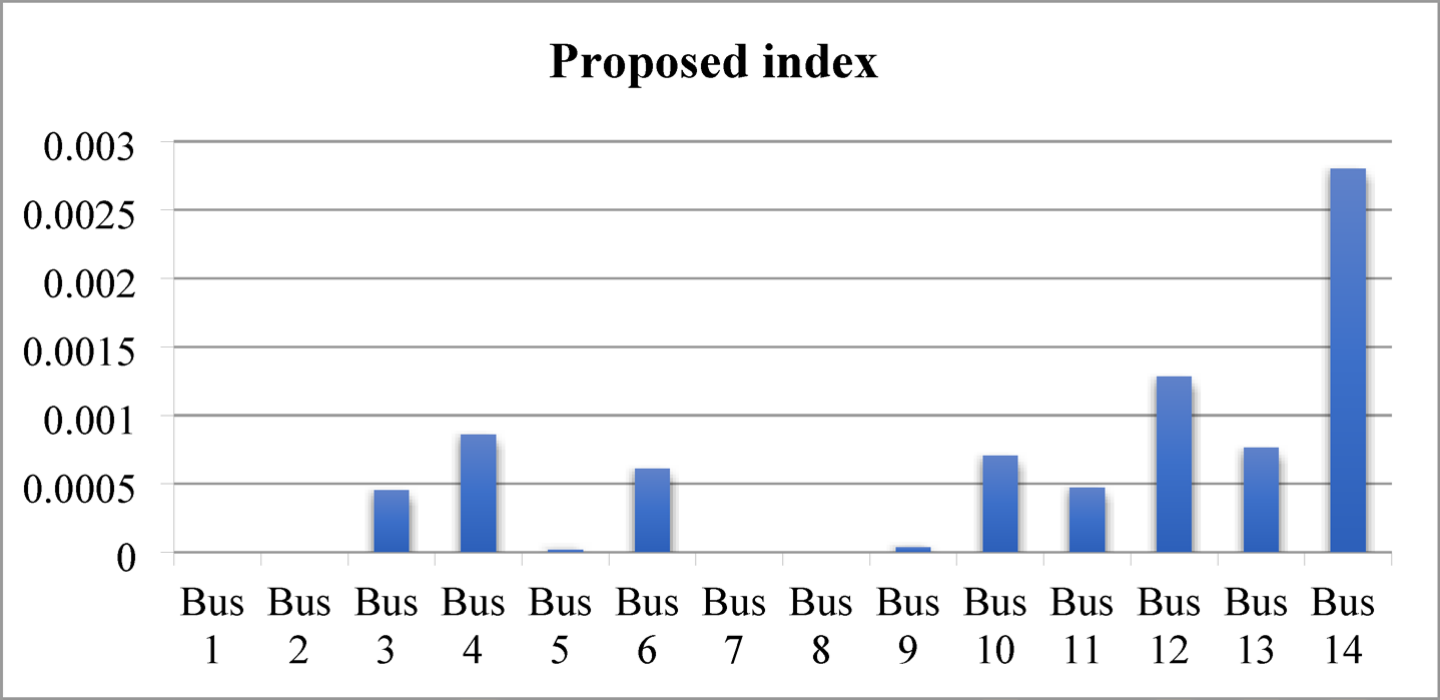
Ranking of proposed index and voltage sensitivity.

When the load flow is executed at a 200% loading level, divergence occurs. Therefore, the loading is gradually reduced until load flow convergence is achieved at 190% loading. At this operating condition, the voltage magnitudes at several buses, prior to compensation, fall below the permissible limits, as shown in Fig. 19. The proposed index is then applied to the test network and identifies bus 14 as the weakest bus requiring compensation that is shown in Fig. 20. To restore the voltage magnitude at bus 14 to 1.0 p.u., an increase of 19.9% (from 0.801 p.u. to 1.0 p.u.) is required by installing SVC at this bus. The required SVC rating, determined using the sensitivity-based approach, is 63.2 MVAr as follows:

$$\:\:{\frac{dv}{dQ}}_{\mid bus5}=\:0.0030205696\:\frac{\mathrm{p}.\mathrm{u}.}{\mathrm{M}\mathrm{V}\mathrm{A}\mathrm{r}}\:,\:\mathrm{d}\mathrm{v}=0.1909\:\mathrm{p}.\mathrm{u}.,\:\:\:\mathrm{t}\mathrm{h}\mathrm{e}\mathrm{n}\:$$

$$\:\mathrm{d}\mathrm{Q}=\:{\frac{dQ}{dv}}_{\mid bus5}\mathrm{x}\:\:\mathrm{d}\mathrm{v}=\frac{1}{0.0030205696}\:\mathrm{x}0.1909=63.2\:\mathrm{M}\mathrm{V}\mathrm{A}\mathrm{r}$$

The standard SVC rating is selected as 65MVAr according to the manufacture.

**Fig. 21:**
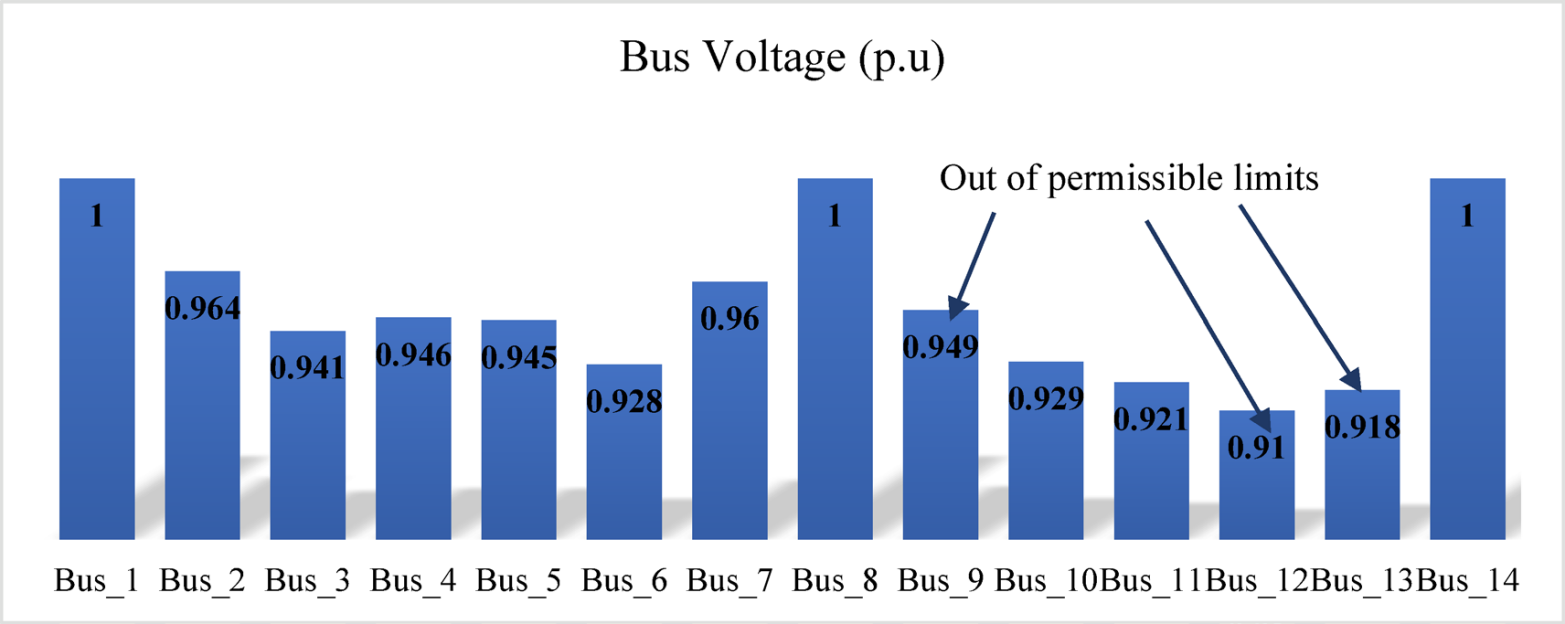
Bus voltages for modified IEEE 14-bus after compensation of bus (14).

**Fig. 22:**
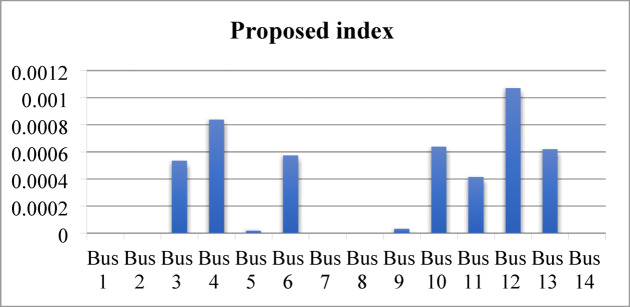
Ranking of proposed index and voltage sensitivity.

Subsequently, the loading level is restored to 200%, and the SVC installed at bus 14 is re-rated using the sensitivity-based approach to satisfy the new operating condition, resulting in a required rating of 17.6 MVAr to maintain the voltage at 1.0 p.u. Despite this compensation, the voltage magnitudes at several buses remain below the permissible limits, as shown in Fig. 21. The proposed index is then applied to identify bus 12 as the weakest bus under the 200% loading condition, as shown in Fig. 22. Accordingly, SVC is installed at bus 12 to increase the voltage magnitude from 0.91 p.u. to 1.0 p.u., with a required rating of 32.5 MVAr determined using sensitivity analysis. Following this compensation, the IPVSI value at bus 14 becomes zero due to the injection of capacitive reactive power by the SVC installed at bus 14.

**Fig. 23:**
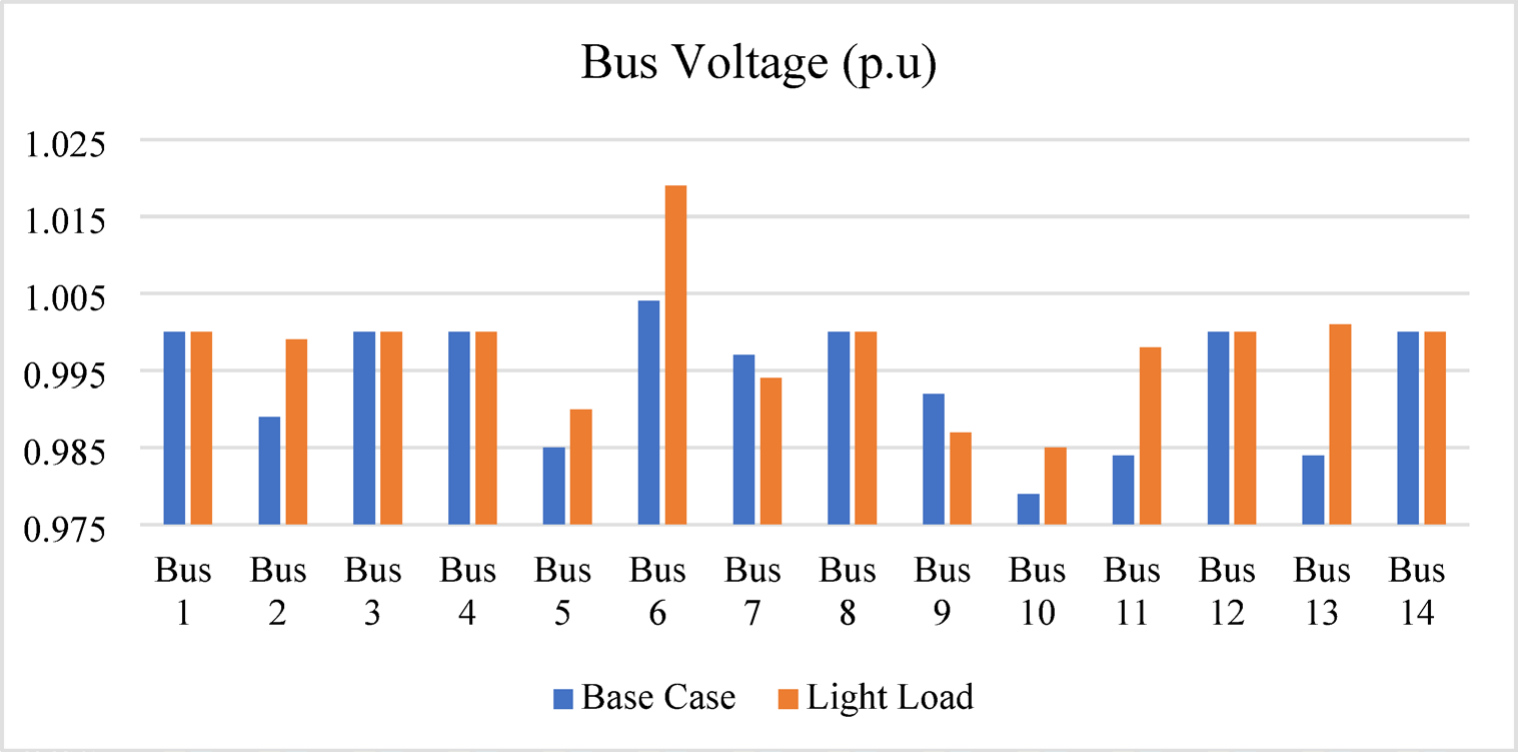
Bus voltages for modified IEEE 14-bus after compensation.

After compensating bus 12, the voltage magnitudes at buses 3 and 10 remain below the permissible limits. Therefore, the proposed index is reapplied to identify the next weakest bus requiring compensation. The IPVSI identifies bus 4 as the weakest bus. Consequently, the voltage magnitude at bus 4 is increased from 0.95 p.u. to 1.0 p.u. by installing SVC with a rating of 70.5 MVAr, as determined using the sensitivity-based approach. All bus voltage magnitudes remain within the acceptable limits, with the minimum voltage of 0.96 p.u. occurring at bus 10 after compensation at buses 14, 12, and 4. Furthermore, the light-load operating condition, examined at 40% loading, confirms that all bus voltages remain within permissible limits under both normal and light-load conditions, as also shown in Fig. 23.

#### (N-1) contingency analysis

The contingency analysis (N–1) is performed for all overhead transmission lines and generator units. The load flow successfully converges for all line contingency cases. The list and numbering of the contingency scenarios are provided in Table 6.

**Fig. 24:**
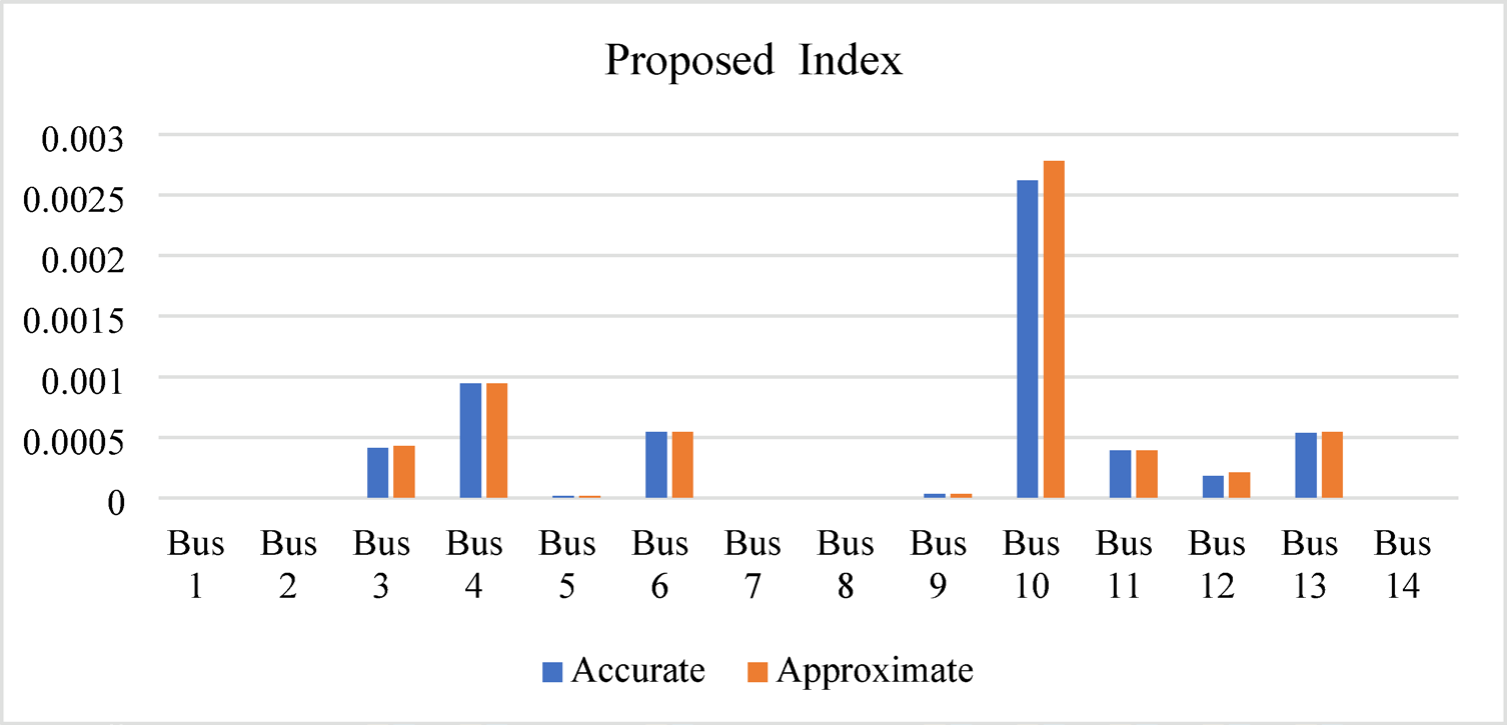
Bus voltages for modified IEEE 14-bus under line contingency (9–10).

The minimum voltage is 0.88 p.u. at bus 10 during the contingency of line (9–10). The proposed index is then re-applied and identifies bus 10 as the weakest bus using both the exact and approximate formulations, as shown in Fig. 24. The difference between the accurate and approximate IPVSI values at bus 10 (0.002620037 and 0.002782167, respectively) is negligible, confirming that either formulation can be reliably used under contingency conditions.

**Fig. 25:**
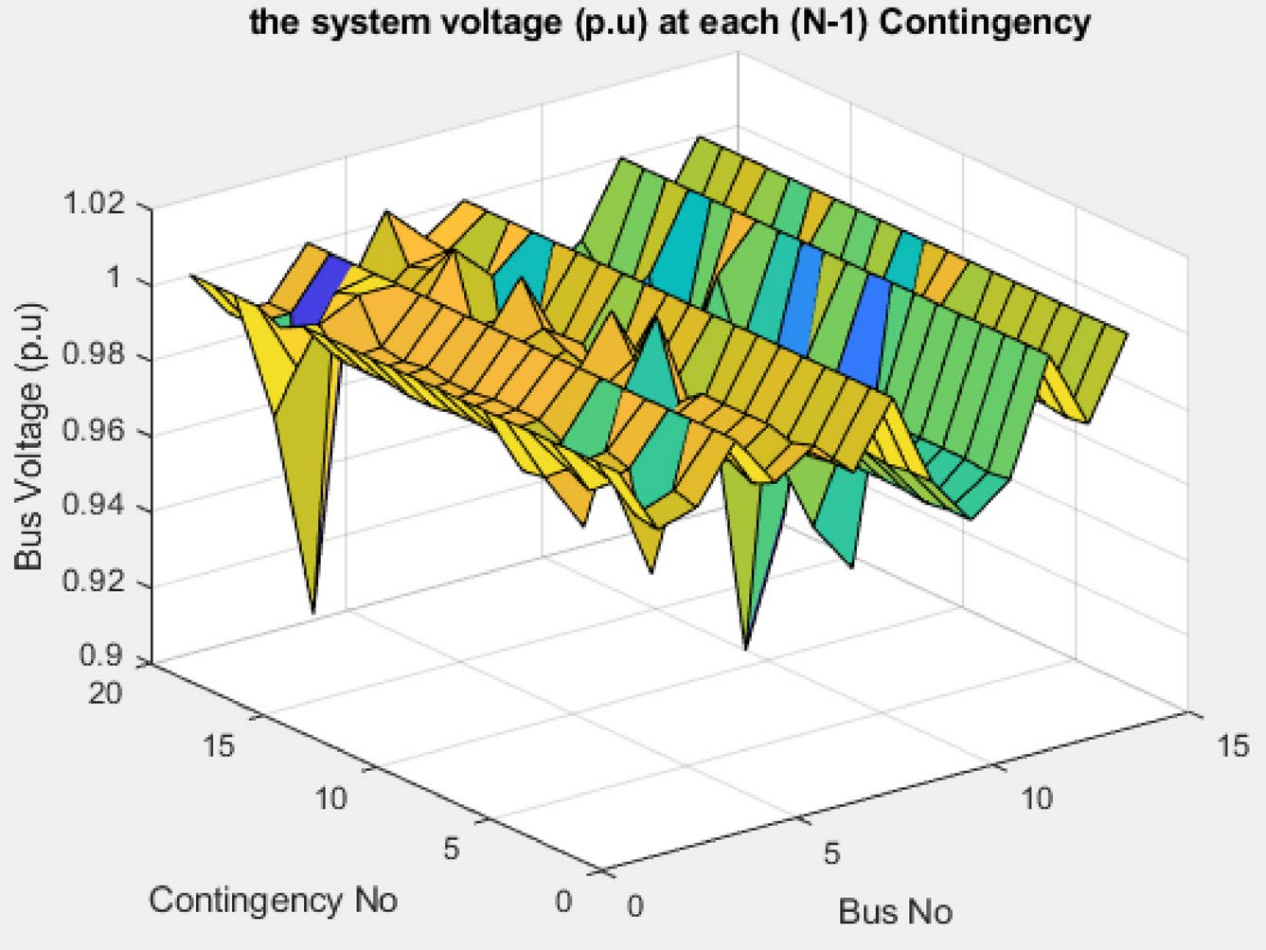
Bus voltages for modified IEEE 14-bus during contingency cases.

However, in this case, the installation of a fixed capacitor is recommended instead of an SVC to reduce the overall cost, since only a limited voltage improvement from 0.88 p.u. to the minimum acceptable value of 0.9 p.u. is required under contingency conditions. The required capacitor rating, determined using sensitivity analysis is five MVAr. Under all contingency conditions, the bus voltage magnitudes remain within the permissible limits (± 10%), as shown in Fig. 25.

**Table 6:** Contingencies cases numbering of modified 14-bus system.

Contingency Case	Line_1_2/1	Line 1_ 5	Line 2_3	Line 2_4	Line 2_5	Line 3_4	Line 4_5	Line 6_11	Line 6_12	Line 6_13
Case **No**	1	2	3	4	5	6	7	8	9	10
Contingency Case	Line 9_10	Line 9_14	Line 13_14	Line10_11	Line 12_13	Wind Generator at bus 1	PV System at bus 2	Wind Generator at bus 3	PV System at bus 6	Hydro generation at bus 8
Case **No**	11	12	13	14	15	16	17	18	19	20

The final RPP results are summarized in Table 7. The maximum required rating of SVC (1), installed at bus 4, is 94.4 MVAr, which occurs during the contingency of wind generator at bus 3. Meanwhile, the maximum required rating of SVC (2) at bus 12 is 22.3 MVAr during line (6–12) contingency, and the maximum required rating of SVC (3) at bus 14 is 47.6 MVAr during the line contingency (6–13).

#### Results comparison

To evaluate the performance of the proposed RPP methodology, metaheuristic optimization algorithms^32^ are employed to minimize the voltage deviation profile as a single objective function, as defined in Eqs. (22) and (23). The locations and ratings of the SVCs are treated as control variables and are encoded within the search agents, as expressed in Eq. (24), to determine the optimal allocation and sizing of SVCs while satisfying the previously stated constraints. PSO^33^ and GWO^34^ are adopted as benchmark algorithms for comparison, as they are widely used in RPP studies.

22 $$\:Voltage\:Profile=\sum\:_{i=1}^{{N}_{b}}{({Vset}_{i}-{V}_{b})}^{2}$$

23 $$\:{Vset}_{i}=\left\{\begin{array}{c}{V}_{set}\:\:\:\:\:\:\:\:\:for\:controlled\:bus\:\\\:1\:\:\:\:\:\:\:\:\:\:\:\:\:\:\:\:\:\:\:\:\:for\:P-Q\:bus\end{array}\right.$$

24 $$\:\mathrm{S}\mathrm{e}\mathrm{a}\mathrm{r}\mathrm{c}\mathrm{h}\:\mathrm{a}\mathrm{g}\mathrm{e}\mathrm{n}\mathrm{t}=\left[{location}_{1}\:\:\:\:\:..\dots\:{location}_{Nsvc\:,}\:\:{Q}_{SVC1}\dots\:..{Q}_{SVCN\:}\:\right]\:\:\:\:\:\:\:\:\:\:\:\:\:\:\:\:\:\:\:\:\:\:$$

Where $\:{N}_{b}$ is the number of buses, $\:{V}_{b}$ is the evaluated voltage at bus *i*, $\:{Vset}_{i}$ is the voltage setting at bus *i*.

Table 7 presents the optimal SVC locations and ratings obtained using the proposed RPP methodology, PSO, and GWO. The SVC sizes represent the reactive power requirements calculated directly from the optimization results. It is observed that buses 14 and 4 are consistently selected by all optimization methods, indicating that these weak buses exert a dominant influence on the overall system voltage profile.

**Table 7:** Final RPP results of modified IEEE 14-bus system.

RPP Methodology	Proposed RPP	PSO	GWO
SVCs Locations and Sizing (MVAr)	$\:{Q}_{SVC}$(@bus 4) = 94.4 (Capacitive) $\:{Q}_{SVC}$(@bus 12) = 22.3 (Capacitive) $\:{Q}_{SVC}$(@bus 14) = 47.6 (Capacitive) $\:{Q}_{Capacitor}$ (@bus 10) = 5	$\:{Q}_{SVC}$(@bus 4) = 94 (Capacitive) $\:{Q}_{SVC}$(@bus 10) = 31 (Capacitive) $\:{Q}_{SVC}$(@bus 14) = 50.5 (Capacitive)	$\:{Q}_{SVC}$(@bus 4) = 94.6 (Capacitive) $\:{Q}_{SVC}$(@bus 11) = 28.3 (Capacitive) $\:{Q}_{SVC}$(@bus 14) = 54.5 (Capacitive)
SVCs Locations and final ratings (MVAr)	$\:{Q}_{SVC}$(@bus 4) = 95 (Capacitive) $\:{Q}_{SVC}$(@bus 12) = 25 (Capacitive) $\:{Q}_{SVC}$(@bus 14) = 50 (Capacitive) $\:{Q}_{Capacitor}$ (@bus 10) = 5	$\:{Q}_{SVC}$(@bus 4) = 95 (Capacitive) $\:{Q}_{SVC}$(@bus 10) = 35 (Capacitive) $\:{Q}_{SVC}$(@bus 14) = 55 (Capacitive)	$\:{Q}_{SVC}$(@bus 4) = 95 (Capacitive) $\:{Q}_{SVC}$(@bus 11) = 30 (Capacitive) $\:{Q}_{SVC}$(@bus 14) = 55 (Capacitive)

Table 8 provides a comprehensive performance comparison based on several quantitative indices, including the voltage profile, total line losses, annual savings (Eq. 25) and investment cost (Eq. 21). Collectively, these performance metrics clearly demonstrate the effectiveness and economic viability of the proposed RPP methodology.

25 $$\:{\:\:\:\:\:\:\:\:\:\:Cost}_{save}=\left({P}_{loss}^{init}-{P}_{loss}^{after}\right)\times\:D\times\:TAF$$

$$\:{P}_{loss}^{init},\:{P}_{loss}^{after}$$ are the line losses before and after the compensation respectively,$$\:{\:Cost}_{save}\:$$is the annual operational cost saving, D is the annual duration in hours (8760 h) and $$\:TAF$$ relates to the tariff rate ($/MWh) is taken 164 $/MWh^35^.

The proposed RPP approach improves the voltage profile by 98.93%, outperforming PSO (98.79%) and GWO (98.75%). In addition, the proposed RPP achieves a lower investment cost of USD 17 million compared to the other methods, while PSO yields a slightly higher annual saving (by approximately USD 0.201 million) than the proposed RPP.

**Table 8:** Comprehensive performance comparison for modified IEEE 14-bus system.

RPP Methodology	Proposed RPP	PSO	GWO
Line Losses (MW)	18.5144	18.3752	18.5402
Voltage Profile (p.u.)	0.002138	0.002416	0.002508
Annual Saving (M$/year)	1.705	1.906	1.669
Investment Cost (M$)	17	18.5	18

### Modified IEEE 39-Bus test system

**Fig. 26:**
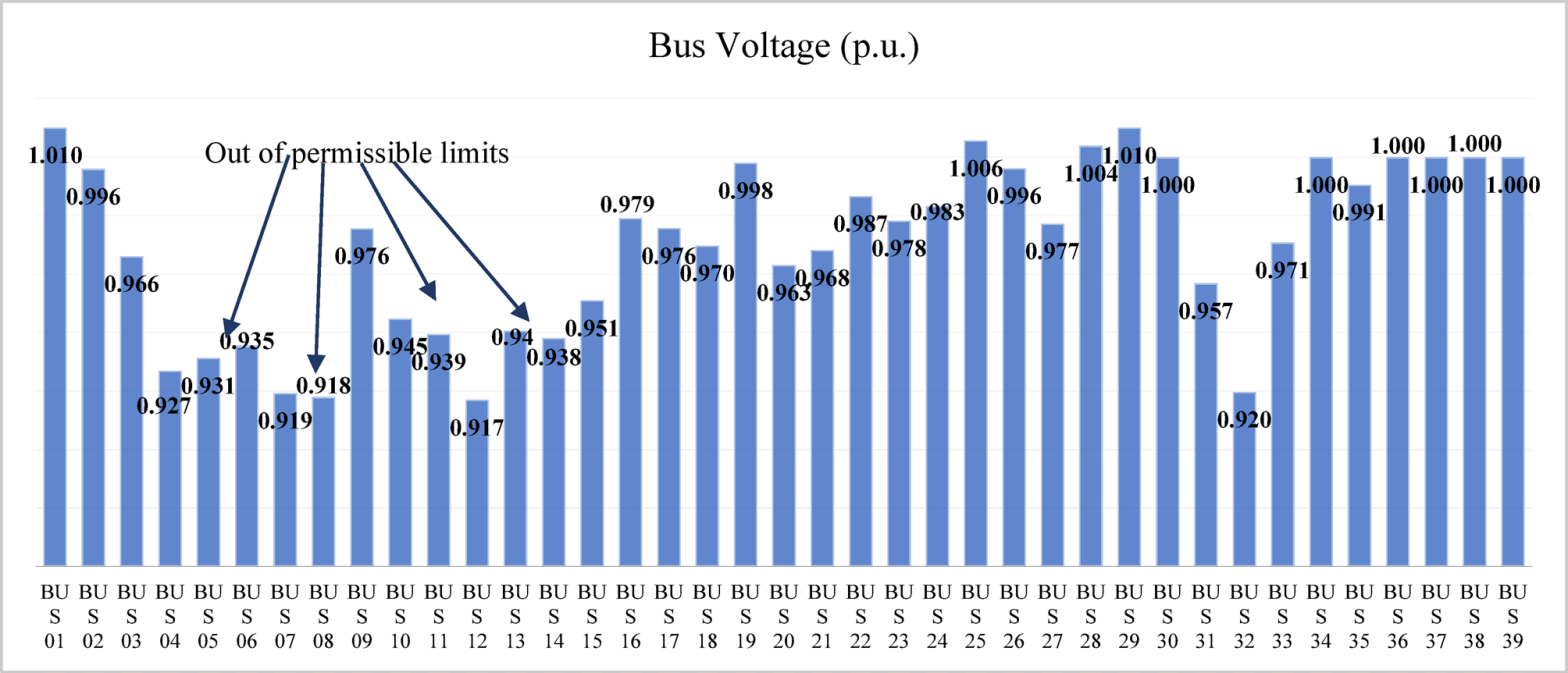
Bus voltages for modified IEEE 39-bus before compensation.

The modified IEEE 39-bus test system retains the standard line and transformer parameters of the original network, as shown in Fig. 4; however, the loads are increased by 135% to represent a heavily stressed operating condition. Generators 3, 4, 6, and 9 are replaced with renewable energy resources, and the generation data of these resources are summarized in Table 9. Both lines (16–19) and (15–16) are considered as double line circuits to prevent the islanding conditions. Bus voltage analysis indicates that several buses have voltages below the permissible limit, specifically buses 4–8, 10–14, and 32 as shown in Fig. 26.

**Table 9:** Generation data of 39-bus system.

Gen Type	Busbar	Bus T.	Voltage Mag. (*p*.u.)	Voltage Angle (deg)	Rated Power (MVA)	Lower Reactive Power Limit (MVAr)	Upper Reactive Power Limit (MVAr)	Active Power (MW)
G1 (Synch) (Two units)	Bus 39	SL	1	0	5000	−1550	2600	1820
G2 (Synch)	Bus 31	PV	1	---	700	−217	364	540
G3 (PV)	Bus 32	PV	1	---	1000	−310	310	877
G4 (Wind)	Bus 33	PV	1	---	900	−279	279	810
G5 (Synch) (Two units)	Bus 34	PV	1	---	450	−139	234	686
G6 (PV)	Bus 35	PV	1	---	1000	−310	310	877
G7 (Synch)	Bus 36	PV	1	---	750	−232	390	675
G8 (Synch)	Bus 37	PV	1	---	800	−248	416	729
G9 (Wind)	Bus 38	PV	1	---	1200	−372	372	945
G10 (Synch)	Bus 30	PV	1	---	1000	−310	520	337

**Fig. 27:**
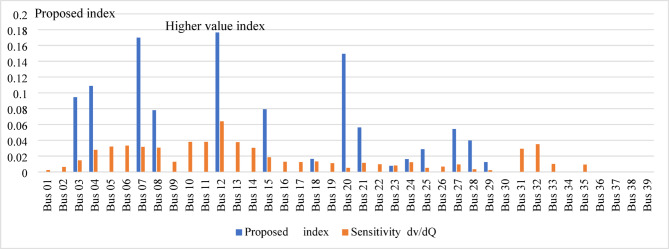
Ranking of the proposed index and the voltage sensitivity.

**Fig. 28:**
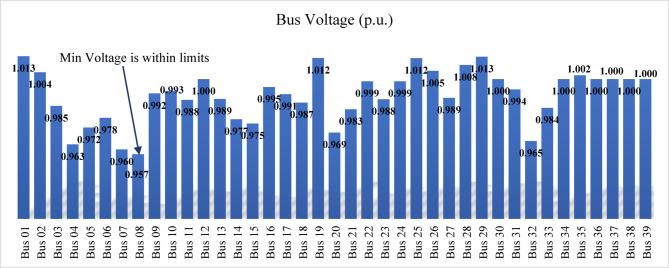
Bus voltages for modified IEEE 39-bus after compensation bus (12).

The proposed index is then applied to the test network, identifying bus 12 as the candidate bus with the highest index value and higher voltage sensitivity that is shown in Fig. 27. The voltage at bus 12 before compensation is 0.917 p.u. and should be increased to 1.0 p.u. To achieve this, SVC is installed at bus 12, with its rating determined using the sensitivity approach as 145 MVAr. After compensation, the minimum voltage at bus 12 is 0.96 p.u., which is within the permissible limits as shown in Fig. 28.

#### (N-1) contingency analysis

**Fig. 29:**
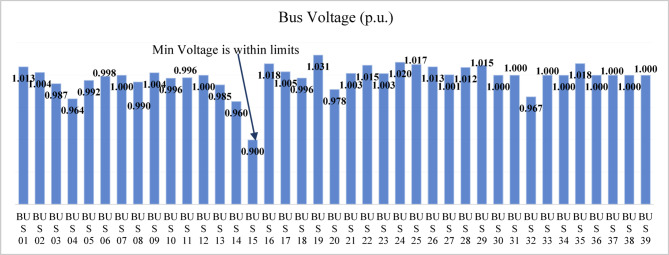
Bus voltages for modified IEEE 39-bus after compensation of bus (7) during line (15–16) contingency.

The contingency analysis (N-1) is conducted for all overhead transmission lines and generator units. During the line (15–16) contingency, the voltage at bus 15 drops to 0.85 p.u., which is below the permissible limit. The proposed index is applied to identify bus 7 as the weakest bus for compensation, ensuring that the minimum voltage during this contingency reaches 0.9 p.u. at bus 15 that is shown in Fig. 29. The SVC rating, determined using the sensitivity approach, is 375 MVAr.

**Fig. 30:**
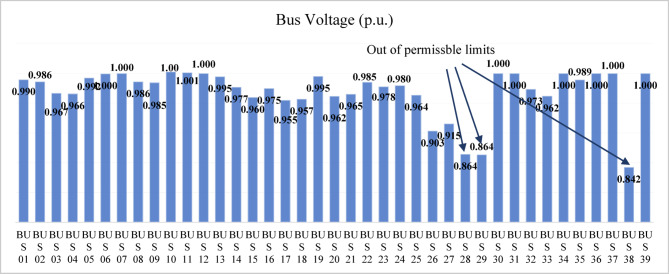
Bus voltages for modified IEEE 39-bus during wind generation (G9) contingency.

The forced outage of the wind generator (G9) at bus (38) causes the voltage at the bus to drop below the permissible limit that is shown in Fig. 30. The proposed index is applied to select bus 29, which has the highest index value among all buses, as the weakest bus for compensation. Using the sensitivity approach, the SVC rating is calculated to raise the voltage from 0.86 p.u. to 0.923 p.u., ensuring that the minimum voltage at bus 38 reaches 0.9 p.u. during G9 contingency. The resulting SVC rating is 81 MVAr (capacitive).

The light-load case, studied at 40% loading, results in an overvoltage at bus 19, with a voltage level of 1.059 p.u. To mitigate this, an inductive SVC is installed to absorb the excess capacitive reactive power. The SVC rating, determined using the voltage sensitivity approach, is 37 MVAr (inductive), which reduces the voltage at bus 19 from 1.059 p.u. to 1.05 p.u., keeping it within the permissible limits.

**Fig. 31:**
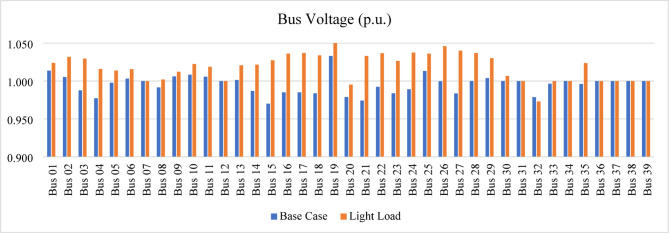
Bus voltages for modified IEEE 39-bus during normal and light loading.

**Fig. 32:**
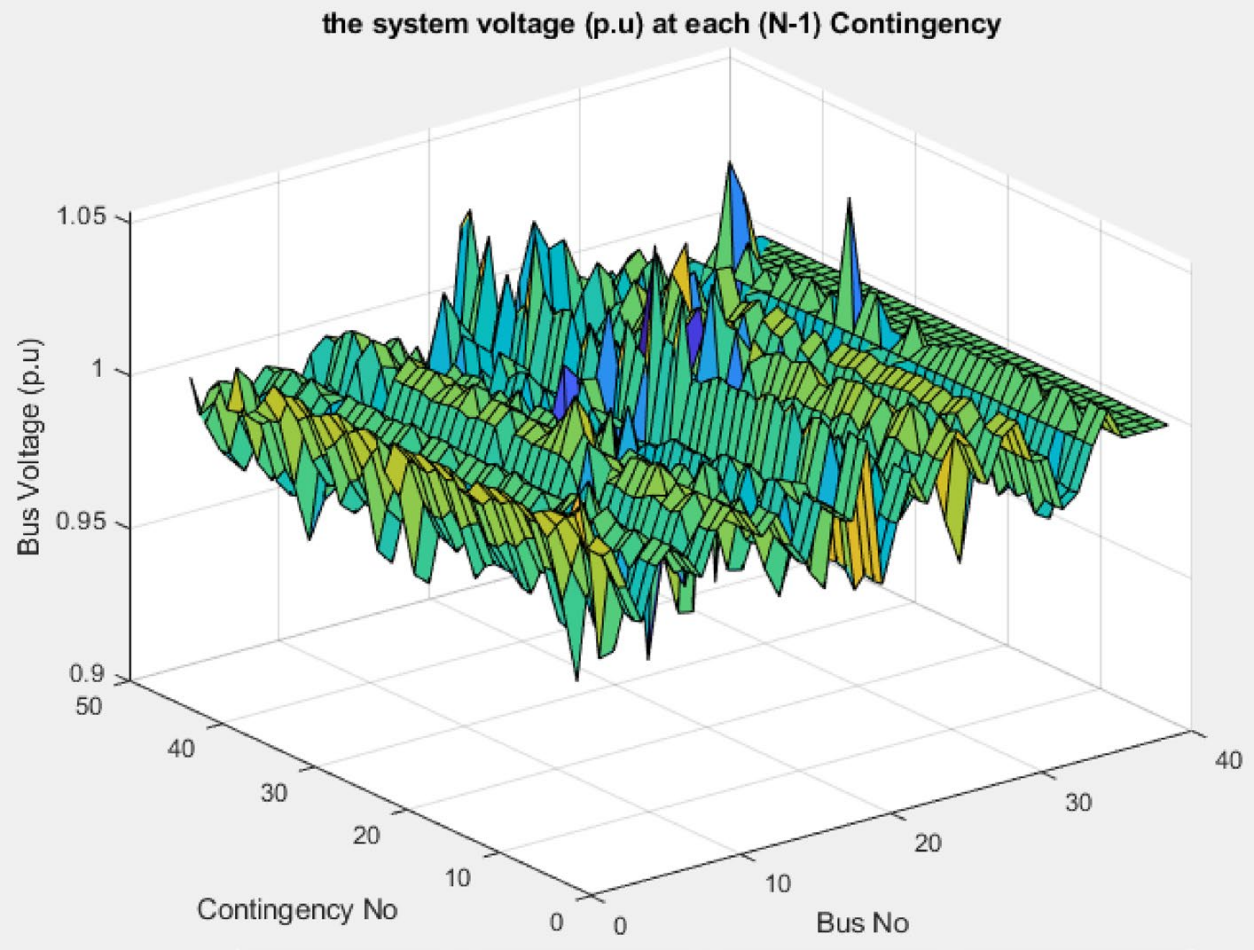
Bus voltages for modified IEEE 39-bus during contingency (N-1).

The bus voltage results for both normal operation and light-load conditions after the compensation at bus 19 are shown in Fig. 31. All bus voltages remain within ± 10% limits during all line and generator unit contingency cases, with the minimum voltage observed as 0.9 p.u. at bus 21 during the line (21–22) and G9 contingencies as shown in Fig. 32. The (N-1) contingency cases and their numbering are presented in Table 10.

**Table 10:** Contingencies cases numbering of modified 39-bus system.

Contingency Case	Line 01–02	Line 01–39	Line 02–03	Line 02–25	Line 03–04	Line 03–18	Line 04–05	Line 04–14	Line 05–06	Line 05–08
Case **No**	1	2	3	4	5	6	7	8	9	10
Contingency Case	Line 06–07	Line 06–11	Line 07–08	Line 08–09	Line 09–39	Line 10–11	Line 10–13	Line 13–14	Line 14–15	Line 15–16
Case **No**	11	12	13	14	15	16	17	18	19	20
Contingency Case	Line 16–17	Line 16–19	Line 16–21	Line 16–24	Line 17–18	Line 17–27	Line 21–22	Line 22–23	Line 23–24	Line 25–26
Case **No**	21	22	23	24	25	26	27	28	29	30
Contingency Case	Line 26–27	Line 26–28	Line 26–29	Line 28–29	G1	G2	G3	G4	G5	G6
Case **No**	31	32	33	34	35	36	37	38	39	40
Contingency Case	G7	G8	G9	G10						
Case **No**	41	42	43	44						

#### Results comparison

The final RPP results are summarized in Table 11. The proposed RPP methodology yields the maximum required SVC ratings for devices SVC-1, SVC-2, and SVC-3 at buses 12, 7, and 29 under the G9 contingency condition, with corresponding capacitive ratings of 67 MVAr, 400 MVAr, and 81 MVAr, respectively. In contrast, the maximum required rating for SVC-4, installed at bus 19, is 37 MVAr (inductive), which occurs under the light-load operating condition.

**Table 11:** Final RPP results of modified IEEE 39-bus system.

RPP Methodology	Proposed RPP	PSO	GWO
SVCs Locations and Sizing (MVAr)	$\:{Q}_{SVC}$(@bus 7) = 400 (Capacitive) $\:{Q}_{SVC}$(@bus 12) = 67 (Capacitive) $\:{Q}_{SVC}$(@bus 19)=−37 (Inductive) $\:{Q}_{SVC}$(@bus 29) = 81 (Capacitive)	$\:{Q}_{SVC}$(@bus 8) = 438 (Capacitive) $\:{Q}_{SVC}$(@bus 12) = 74 (Capacitive) $\:{Q}_{SVC}$(@bus 19)=−37 (Inductive) $\:{Q}_{SVC}$(@bus 26) = 153 (Capacitive)	$\:{Q}_{SVC}$(@bus 7) = 412 (Capacitive) $\:{Q}_{SVC}$(@bus 15) = 79 (Capacitive) $\:{Q}_{SVC}$(@bus 19)=−37 (Inductive) $\:{Q}_{SVC}$(@bus 28) = 84 (Capacitive)
SVCs Locations and final ratings (MVAr)	$\:{Q}_{SVC}$(@bus 7) = 400 (Capacitive) $\:{Q}_{SVC}$(@bus 12) = 70 (Capacitive) $\:{Q}_{SVC}$(@bus 19)=−40 (Inductive) $\:{Q}_{SVC}$(@bus 29) = 85 (Capacitive)	$\:{Q}_{SVC}$ (@bus 8) = 440 (Capacitive) $\:{Q}_{SVC}$(@bus 12) = 75 (Capacitive) $\:{Q}_{SVC}$(@bus 19)=−40 (Inductive) $\:{Q}_{SVC}$(@bus 26) = 155(Capacitive)	$\:{Q}_{SVC}$(@bus 7) = 415 (Capacitive) $\:{Q}_{SVC}$(@bus 15) = 80 (Capacitive) $\:{Q}_{SVC}$(@bus 19)=−40 (Inductive) $\:{Q}_{SVC}$(@bus 28) = 85 (Capacitive)

As shown in Table 12, the proposed RPP approach improves the voltage profile by 93.31%, which is nearly identical to the performance of PSO (93.42%), with a negligible difference of 0.11% (equivalent to 0.00008 p.u.). Moreover, the proposed RPP achieves a lower investment cost of USD 58.5 million compared with the other methods. GWO results in a slightly higher annual saving (by approximately USD 0.464 million) than the proposed RPP.

**Table 12:** Comprehensive performance comparison for modified IEEE 39-bus system.

RPP Methodology	Proposed RPP	PSO	GWO
Line Losses (MW)	46.1177	45.9302	45.7947
Voltage Profile (p.u.)	0.004849	0.004769	0.004972
Annual Saving (M$/year)	6.406	6.675	6.870
Investment Cost (M$)	58.5	71	62

## Conclusion

In this paper, RPP is carried out using a proposed voltage stability index to allocate reactive power compensators under normal operating conditions, lines and generators (*N* − 1) contingencies, light-load conditions, and system configurations that integrate both conventional and renewable energy sources. The proposed stability index is validated using the standard IEEE 9, 14, and 39-bus test systems. Consistent with voltage stability indices reported in the literature, the proposed index identifies buses 5, 14, and 12 as the weakest buses in the IEEE 9, 14, and 39-bus systems, respectively.

The main contribution of the proposed Index lies in its simplified mathematical expression, ease of implementation in computational programs, and elimination of the need for multiple load-flow solutions. Based on the IPVSI, a sequential reactive RPP approach is developed to identify weak buses for SVC placement. A sensitivity-based method is then applied to determine the SVC ratings. The proposed sequential RPP strategy successfully achieves the minimum compensator capacity required to maintain all bus voltages within permissible limits.

The proposed RPP methodology is further evaluated on modified IEEE 14- and 39-bus systems incorporating renewable energy resources. The results indicate that three SVCs and one fixed capacitor are required for the modified IEEE 14-bus system, while four SVCs are sufficient for the modified IEEE 39-bus system. An extensive comparative analysis is conducted against PSO and GWO techniques to assess voltage profile improvement and economic performance.

Overall, although metaheuristic optimization algorithms are capable of achieving comparable voltage profile enhancements, they generally involve higher computational burden and more extensive feasibility analyses. In contrast, the proposed RPP methodology offers a structured and sequential planning framework that achieves improved voltage profiles with minimum investment cost and reduced computational complexity. Nevertheless, when multiple or alternative optimization objectives are required, metaheuristic algorithms may provide greater flexibility and are therefore more appropriate.

## Data Availability

All data generated or analyzed during this study are included in this article. The datasets used and generated during the current study are available from the corresponding author upon reasonable request.
